# Imaging flow cytometry with a real-time throughput beyond 1,000,000 events per second

**DOI:** 10.1038/s41377-025-01754-9

**Published:** 2025-02-10

**Authors:** Jiehua Zhou, Liye Mei, Mingjie Yu, Xiao Ma, Dan Hou, Zhuo Yin, Xun Liu, Yan Ding, Kaining Yang, Ruidong Xiao, Xiandan Yuan, Yueyun Weng, Mengping Long, Taobo Hu, Jinxuan Hou, Yu Xu, Liang Tao, Sisi Mei, Hui Shen, Yaxiaer Yalikun, Fuling Zhou, Liang Wang, Du Wang, Sheng Liu, Cheng Lei

**Affiliations:** 1 The Institute of Technological Sciences, Wuhan University, Wuhan, 430072 China; 2 School of Computer Science, Hubei University of Technology, Wuhan, 430068 China; 3 Division of Materials Science, Nara Institute of Science and Technology, Takayama-cho, 8916-5 Japan; 4 School of Science, Hubei University of Technology, Wuhan, 430068 China; 5 Department of Pathology, Peking University Cancer Hospital, Beijing, 100142 China; 6 Department of Breast Surgery, Peking University People’s Hospital, Beijing, 100044 China; 7 Department of Thyroid and Breast Surgery, Zhongnan Hospital, Wuhan University, Wuhan, 430071 China; 8 Department of Radiation and Medical Oncology, Zhongnan Hospital, Wuhan University, Wuhan, 430071 China; 9 People’s Hospital of Anshun City Guizhou Province, Anshun, 561000 China; 10 Department of Hematology, Zhongnan Hospital, Wuhan University, Wuhan, 430071 China; 11 National Engineering Laboratory for Next Generation Internet Access System, School of Optics and Electronic Information, Huazhong University of Science and Technology, Wuhan, 430074 China; 12 Suzhou Institute of Wuhan University, Suzhou, 215000 China; 13 Shenzhen Institute of Wuhan University, Shenzhen, 518057 China

**Keywords:** Imaging and sensing, Optofluidics

## Abstract

Imaging flow cytometry (IFC) combines the imaging capabilities of microscopy with the high throughput of flow cytometry, offering a promising solution for high-precision and high-throughput cell analysis in fields such as biomedicine, green energy, and environmental monitoring. However, due to limitations in imaging framerate and real-time data processing, the real-time throughput of existing IFC systems has been restricted to approximately 1000-10,000 events per second (eps), which is insufficient for large-scale cell analysis. In this work, we demonstrate IFC with real-time throughput exceeding 1,000,000 eps by integrating optical time-stretch (OTS) imaging, microfluidic-based cell manipulation, and online image processing. Cells flowing at speeds up to 15 m/s are clearly imaged with a spatial resolution of 780 nm, and images of each individual cell are captured, stored, and analyzed. The capabilities and performance of our system are validated through the identification of malignancies in clinical colorectal samples. This work sets a new record for throughput in imaging flow cytometry, and we believe it has the potential to revolutionize cell analysis by enabling highly efficient, accurate, and intelligent measurement.

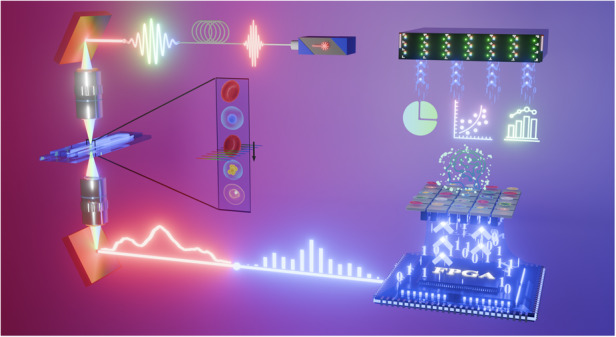

## Introduction

Imaging flow cytometry (IFC) has revolutionized cell analysis across various fields, including biology, microbiology, pharmacology, hematology, and immunology. IFC can capture a large number of cellular images quickly and accurately, providing detailed morphological data to analyse diverse cell populations with high statistical reliability^1–10^. However, traditional IFC systems, which use CCD or CMOS image sensors, face limitations due to their long exposure and readout times, restricting their throughput to around 1000 events per second (eps), which hinders practical applications^4–6,11–18^.

To improve the throughput, Goda et al. introduced optical time-stretch imaging to IFC, pioneering optofluidic time-stretch (OTS) IFC. This innovation increased IFC throughput to over 1,000,000 eps with sub-micron spatial resolution^19,20^, significantly enhancing its capabilities for applications like biomedicine, green energy production, and environmental monitoring^21–29^. However, the high throughput of OTS-IFC generates massive amounts of data, straining transmission and processing systems. As a result, OTS-IFC typically uses a high-speed oscilloscope for data digitization and processes the image data offline on a computer, limiting the system’s actual throughput to around 1,000 eps^25,30^. To explore its real-time imaging capability, Goda et al. developed an OTS-IFC system using an analog-to-digital converter (ADC) and a field-programmable gate array (FPGA)^26^. This system performs initial data processing on the FPGA and selectively transfers data to the onboard memory for further processing, enabling real-time cell analysis at a flow rate of 4 m/s and a spatial resolution of about 1.4 µm, useful for detecting rare cells. Furthermore, Shi et al. designed the coprime line scan super-resolution (CLSS) method^31–33^, combining low-pixel rows to create high-resolution images, achieving a throughput of approximately 10,000 eps.

Despite these advancements, the full potential of OTS-IFC for high-throughput cell analysis remains constrained by bandwidth and data processing limitations. To overcome these challenges, we designed an OTS-IFC system featuring an online ADC and data processor. Using an 80-MHz laser source and a 10 GS/s ADC, the system enables high-speed imaging of cells flowing at rapid rates. Our real-time data processing algorithm efficiently analyzes and filters the data, significantly reducing its volume to align with commercial transmission and storage capacities. This system achieves real-time IFC with a throughput exceeding 1,000,000 eps and a spatial resolution surpassing 780 nm, setting a new benchmark in real-time IFC performance. Additionally, by integrating advanced image analysis algorithms into the data processor, the system demonstrated 99.90% classification accuracy for blood cells and successfully detected rare events. The system was also applied to differentiate tumor from normal tissues in colorectal samples, highlighting its clinical potential. With its ability to perform real-time cell analysis, this OTS-IFC offers a promising solution for various high-throughput, high-precision cell analysis applications, opening new avenues in biomedical research and diagnostics.

## Results

### Configuration of the real-time cytometry

**Fig. 1:**
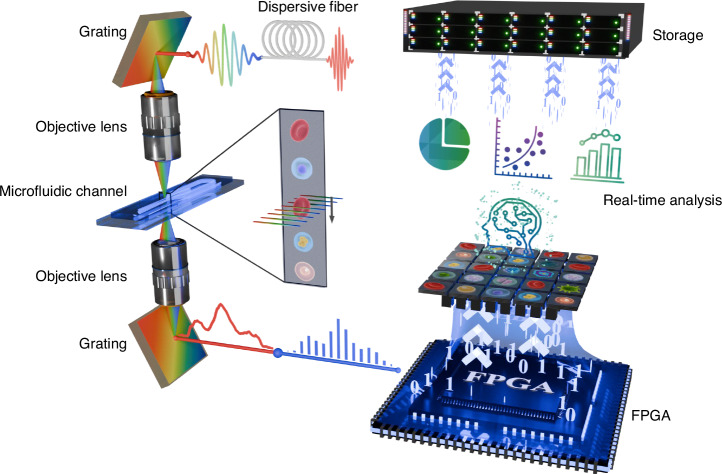
Overview of our optofluidic time-stretch imaging flow cytometry

The schematic of our real-time OTS-IFC is illustrated in Fig. 1. The optical pulses generated from the broadband mode-lock laser (Vitara Modelocked Ti:S Laser, center wavelength: 780 nm, 3-dB bandwidth: 40 nm, repetition rate: 80 MHz) first enter the dispersive fiber (YOFC CS1013-A, GVD: 186 ps/nm), where the pulses are temporally stretched due to the different transmission velocities of different frequency components in the dispersive fiber^34–36^, which means that the light pulse’s different frequencies (colors) are spread out over time as they exit the fiber. Therefore, the temporal waveforms of the pulses are consistent with their spectra as they come out of the fiber. The stretched pulse then passes through the first diffraction grating (Thorlabs GR25-0608, groove density: 600 lines/mm). This grating spatially disperses the different frequency components of the pulse, meaning that different colors of the pulse are directed to different positions. These dispersed colors (frequencies) are focused by the first objective lens (OLYMPUS LCPLN50XIR, magnification: 50×, NA: 0.65) and then illuminate different parts of the cells as they flow through a microfluidic channel. This process effectively maps the spatial information of the cell (where different parts of the cell are) into the spectrum of the light pulse. After interacting with the cell, the light is collected by the second objective lens and recombined by a second diffraction grating (with the same properties as the first). After that, the pulses are sequentially detected by the single-pixel photodetector (Newport 1544-B, spectral range: 500-1630 nm, bandwidth: 12 GHz) and digitized by the high-speed digitizer (Teledyne SP Devices ADQ7DC, sampling rate: 10 GS/s) whose sampling clock is synchronized with the pulse laser (More detail are available in Supplementary S1). Finally, the data is sent to the FPGA for online processing and stored in the commercial solid-state drive (SSD) array. As the cells flow in the channel, each pulse captures one cross-section of the cells. By stacking the 1D cross-section together, 2D images of the cells can be recovered. Supplementary Video S1 showcases the workflow of our real-time cytometry.

### The throughput evaluation of the real-time cytometry

**Fig. 2:**
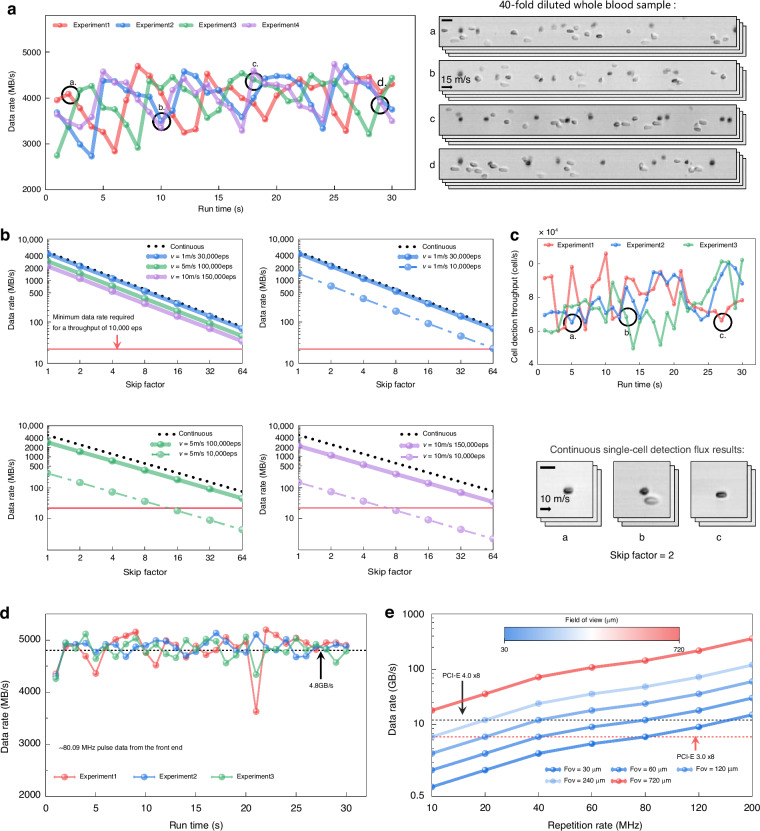
The throughput evaluation of the real-time optofluidic time-stretch imaging flow cytometry. **a** The system’s high DR under extremely high-throughput conditions. **b** The system’s DR reduction under various conditions for single-cell imaging and the impact of applying skip factors. **c** The real-time single-cell detection throughput under high-throughput conditions using a 500-fold diluted blood sample. **d** The DR performance in acquiring all pulse data from the imaging front end. **e** Estimate the DR for continuous cell detection on varying pulse repetition rates and FOV scenarios. Scale bars: 10 µm

In order to evaluate the maximum throughput of the system, we conducted a high-throughput assessment of our cytometry using a 40-fold diluted whole blood sample flowing at a speed of 15 m/s. In experiments characterized by high flow rates and concentrations prone to cell reunions and overlap, we employed large-time window time-stretch imaging to match the evaluation. The results from real-time data rate (DR) experiments and the corresponding image in Fig. 2a demonstrate that when more than 25 cells are imaged within each large-time window time-stretch image within 20 µs, our system achieves a DR of approximately 4000 MB/s, calculated as ~40,960 fps multiplied by 1,600 pulses, each represented by 64 pixels (8-bit). This reflects the system’s capability to detect over 1 million cells per second.

However, it is essential to note that instances of excessive concentration in samples are not commonly encountered in typical clinical applications, where accurate single-cell imaging analysis is often more desirable. In Fig. 2b, we present the system’s DRs under various conditions when performing single-cell imaging, considering different flow rates and throughputs. The figure also illustrates the DR reduction achieved by applying various skip factors, with further details available in Fig. S2 and the “Materials and methods” section. Notably, at a flow rate of 1 m/s and a throughput of 10,000 eps, applying a skip factor of 64 results in an 853-fold reduction in DR. This flexibility is crucial for optimizing the system’s efficiency in real-world clinical scenarios.

In a separate high-throughput assessment, we performed a high-throughput assessment of our cytometry using a 500-fold diluted whole blood sample flowing at a speed of 10 m/s, with the corresponding continuous single-cell detection throughput results presented in Fig. 2c. Over a 30-second experiment, the real-time cell detection throughput ranged from approximately 50,000 to 100,000 cells/s, resulting in DRs varying from about 366 MB/s to 732 MB/s (With a skip factor of 2). Given that the typical concentration of red blood cells (RBCs) in healthy adults falls within the ranges of 4 million/µL to 6 million/µL, and the sample channel flow rate is 14 µL/s at a flow speed of 10 m/s, we can reasonably infer that nearly all cell images within the field of view (FOV) are captured, except for those outside the FOV of the optical system. This implies that we can achieve nearly lossless high-throughput single-cell detection. Supplementary Video S2 proves that the system does not cause loss of events from a data point of view.

In Fig. 2(d), we present three sets of experimental results involving the acquisition of approximately 80.09 MHz pulse data generated from the imaging front end. This data corresponds to a DR of approximately 80.09 MHz pulses × 64 pixels (8-bit), resulting in a throughput of approximately 4888.3 MB/s, as indicated by the highest point on the black dashed line in Fig. 2b. The data is transferred through a PCI-E Gen3 interface and ultimately stored using a multi-thread writing process on a RAID-SSD array. The experimental findings depicted in Fig. 2d demonstrate the stability of the system’s DR, which fluctuates within a small range and consistently hovers around 4.8 GB/s across multiple experiments. These findings indicate that the system’s data transfer and storage mechanisms effectively handle large data volumes without bottlenecks, while precise timing synchronization ensures that image acquisition and data processing are tightly coordinated.

To expand the versatility of our cytometry system for different FOV sizes and pulse repetition rates, Fig. 2e estimates the necessary DRs for continuous detection across varying pulse repetition rates and FOV scenarios. Notably, Fig. 2e illustrates that leveraging the PCIe ×8 Gen4 interface with double DR has the potential to double the field of view for high-throughput continuous cell detection in our cytometry. These estimates are contingent on our assumption that, following spatial domain slit interception, the pulse exhibits a duty cycle of no more than 1/2 and presents a favorable Gaussian profile suitable for imaging. Subsequently, a synchronized FPGA intercepts this pulse in the digital domain, employing a fixed window size of half of the pulse interval. Each sample point presents a spatial resolution of less than 0.8 µm (To satisfy the Nyquist sampling theorem, it is optimal for two sampling points to correspond to one diffraction-limited point), with a consistent data size of 1 Byte (8 bits).

### The cell image quality evaluation of the real-time cytometry

**Fig. 3:**
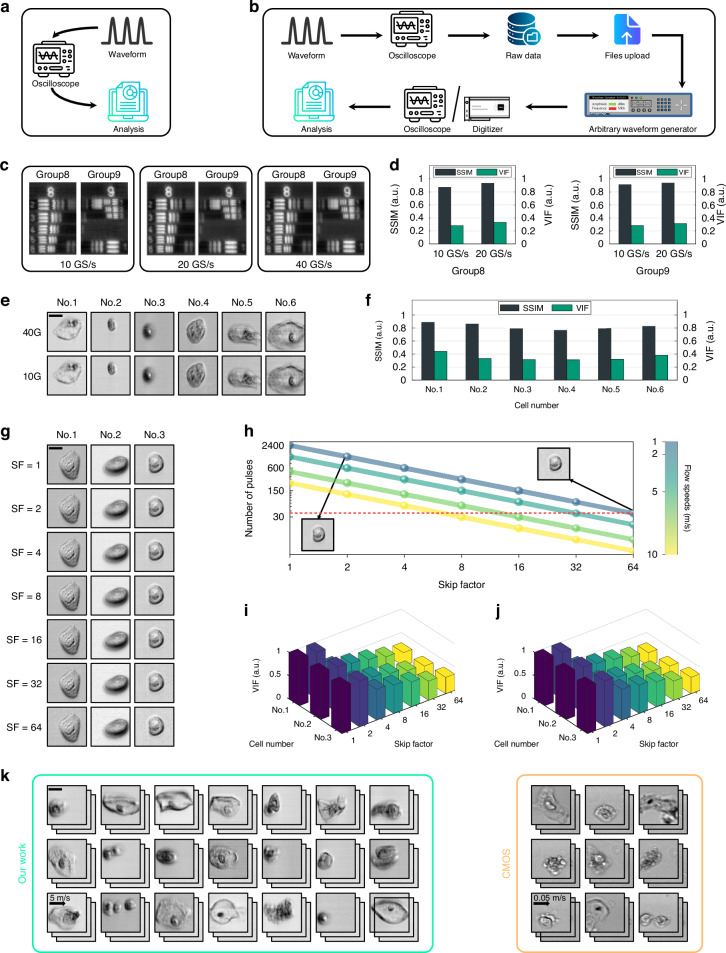
Image quality evaluation of optofluidic time-stretch microscope integrated with the digitizer. **a** The image quality evaluation experimental process of USAF-1951 resolution chart. **b** The image quality evaluation experimental process of cell image. **c**, **d** Images of the group 8 and 9 resolution chart using different sampling rates and the corresponding SSIM and VIF calculation results. **e**, **f** Images of cells at different sampling rates under simulation and the corresponding SSIM and VIF calculation results. **g** Cell images were acquired at different skip factors by the digitizer. **h** The trend of the number of pulses in the cell images was acquired using different skip factors at different flow speeds. **i**, **j** The SSIM and VIF calculation results of the cell images at different skip factors. **k** The cell image libraries of our optofluidic time-stretch imaging flow cytometry and conventional imaging flow cytometry. Scale bars: 10 µm

To mitigate data transfer constraints, we designed the minimum required sampling rate for our optofluidic time-stretch microscopy as 10 GS/s. In validation of this design, as the workflow shown in Fig. 3a, we compared image quality using the USAF-1951 resolution chart. Images were acquired at sampling rates of 10 GS/s, 20 GS/s, and 40 GS/s by leveraging the oscilloscope. As shown in Fig. 3c, images from Groups 8 and 9 of the USAF-1951 resolution chart demonstrate that the 10 GS/s images offer image quality on par with those at 20 GS/s and 40 GS/s. All line pairs of Group 9, Element 3, are distinguishable at all three sampling rates (10 GS/s, 20 GS/s, and 40 GS/s), affirming that our optofluidic time-stretch microscopy achieves a spatial resolution of approximately 780 nm at the 10 GS/s sampling rate, in line with theoretical calculations. Additionally, to demonstrate the equivalence in image quality between cell images acquired at 10 GS/s and 40 GS/s using our optofluidic time-stretch microscopy, we conducted a comparative analysis. As the workflow illustrated in Fig. 3b, we employed an oscilloscope to capture cell image data at a 40 GS/s sampling rate. Subsequently, we harnessed the arbitrary waveform generator (AWG) to create the initial data for the oscilloscope to acquire data at a sample rate of 10 GS/s. The resulting cervical cell image libraries, acquired at these two distinct sampling rates, are presented in Fig. 3e. The images obtained at 10 GS/s clearly exhibit the same cell structures and details as those acquired at 40 GS/s, affirming that our optofluidic time-stretch microscopy maintains its capability to produce high-quality images at the 10 GS/s sampling rate.

Furthermore, we used the image quality index structural similarity (SSIM)^37,38^ index and visual information fidelity (VIF)^39^ as quantitative metrics to assess the image quality acquired by our optofluidic time-stretch microscopy at the 10 GS/s sampling rate and higher sampling rates. Specifically, we utilized the image acquired at 40 GS/s as the reference. We then calculated the SSIM and VIF values of all the other images acquired at lower sampling rates, as depicted in Figs. 3d and f, with the results displayed in the corresponding figures. The SSIM and VIF calculation results for the USAF-1951 images acquired at 10 GS/s closely resemble those obtained at 20 GS/s. Similarly, the SSIM and VIF calculation results for cell images acquired at 10 GS/s mirror those of the USAF-1951 images, indicating a consistently high level of image quality. This underscores the effectiveness of our low sampling rate design in maintaining superior image quality.

To validate the effectiveness of the pulse-skip method (Fig. S2), which is designed to reduce consecutive redundant pulses, as the experimental process illustrated in Fig. 3b, we employed the oscilloscope to acquire cell image data at the sampling rate of 10 GS/s and the flow rate of 1 m/s. We then utilized AWG to generate the original data for the digitizer to acquire at different skip factors. Figure 3g shows three groups of cell images acquired at different skip factors by the digitizer, while Fig. 3(h) shows the trend of the number of pulses in the images after cells are acquired using different skip factors at different flow speeds. As discussed in Fig. S2, the cell image with 30 µm FOV in flow direction should ideally contain at least 38 pulses [the red dotted line in Fig. 3(h)], which means that as the flow speeds increase, the maximum skip factor can be applied will become smaller. As seen in Fig. 3(g), with the increase of skip factors from 1 to 64, the number of pulses contained in each cell image decreases from 2400 to 38 in the situation of 1 m/s, but the cell image quality does not significantly decrease, nor do the details in the image reduce, which indicates the pulse-skip method preserves essential information in cell images. Supplementary Video S3 demonstrates how the system can reduce redundancy by adjusting the parameters.

Similar to above, to further quantitatively assess the image quality of images acquired at different skip factors, we used the image acquired at the skip factor of 1 as the reference and calculated the SSIM and VIF values of all the other images in Fig. 3g, and three groups of cell images calculation results are shown in Fig. 3i) and Fig. 3(j), respectively. As calculation results of three groups of cell images in Fig. 3(i) and Fig. 3(j) show, with the increase of skip factors, the SSIM and VIF calculation results both show a downtrend. However, as long as the chosen skip factor is not close to the theoretically selectable limit value, the calculation results remain at a reasonable level. Especially while the skip factor selected is 2, the SSIM and VIF calculation results are nearly equal to 1, indicating that the image quality is closest to the original. This result suggests that the optimal choice for a skip factor in high-throughput cell detection is 2. Figure 3(k) shows two image libraries of the cervical cells. Images within the blue box are imaged by our cytometry employing the skip factor of 2 at a flow speed of 5 m/s, while the images within the orange box are imaged by a conventional bright-field microscope with a CMOS camera at a flow speed of 0.05 m/s. The images acquired by our cytometry distinctly show the intricate cellular structures with amole contrast at an equivalent level to what a conventional bright-field microscope can achieve, which also indicates that our method of reducing data in each image does not compromise image quality (Further comparisons with CMOS-base IFC at different flow rates are available in Supplementary S3).

### Screening of cervical cells and hematocytes with real-time cytometry

**Fig. 4:**
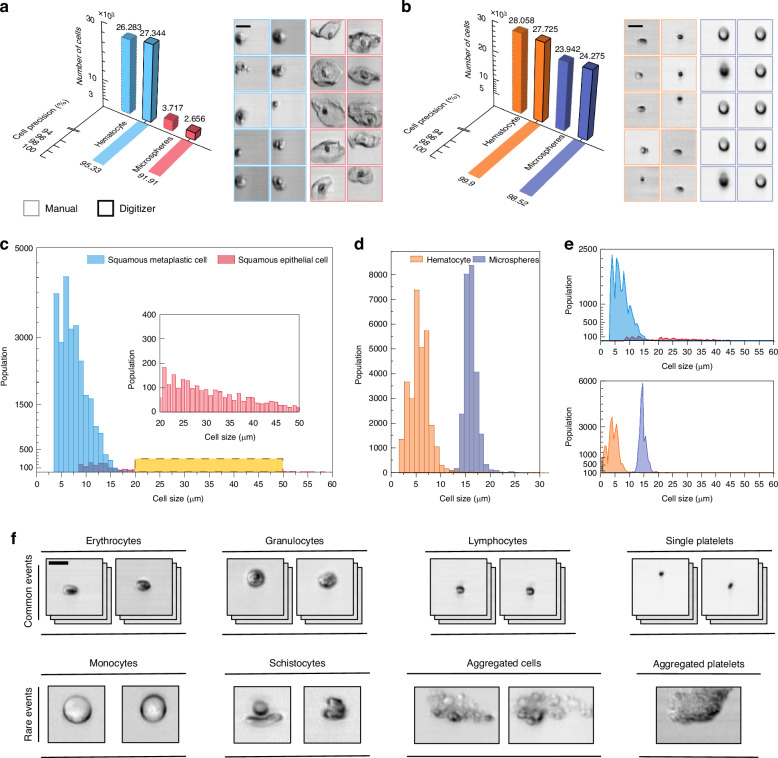
Classification result and detection ability of our optofluidic time-stretch imaging flow cytometry. **a** Classification results of cervical cell images. **b** Classification results of microspheres and whole-blood cells sample images. **c**–**e** The digitizer tags the cell size distribution of the cervical cell sample. **d**, **e** The digitizer tags the cell size distribution of the microspheres and whole-blood cell mixture sample. **f** Some rare events are detected by our optofluidic time-stretch imaging flow cytometry in the single whole blood sample testing. Scale bars: 10 µm

To validate the robustness of our cell size labeling method, we used cell size as a distinguishing feature to classify a dataset of 30,000 cervical cell images. The dataset was divided into squamous metaplastic cells and squamous epithelial cells based on the size labels assigned by the FPGA, using half the field of view of the cell image (15 µm) as the gating threshold. Simultaneously, we manually divide the same set of cell images into two categories to establish a basis for comparison. The classification results are shown in Fig. 4(a). According to the results of manual type, 26068 of 27344 squamous metaplastic cell images are correctly classified, yielding a precision rate of 95.33%, and 2441 of 2656 squamous epithelial cell images are correctly classified, resulting in a precision rate of 91.91%. Figure 4(c) depicts the cell size distribution at 1 µm intervals categorized by the digitizer within the manual classification dataset, while the upper part of Fig. 4(e) illustrates the distribution at 0.5 µm intervals. This screening result shows the potential of our cytometry technique to emerge as a swifter alternative for cervical cancer screening, akin to the ThinPrep Cytologic Test (TCT)^40–42^.

In addition, we also captured 52,000 images from a sample containing a mixture of microspheres (ACME FG15UM-10, 15 µm) and whole-blood cells. Using cell size as a distinguishing feature, the FPGA classified the images into two categories based on a 10 µm gating threshold. Concurrently, we manually categorized the same sample images into two categories as standard for comparison. The classification results are shown in Fig. 4(b). According to the results of manual type, 27699 of 27725 hematocyte images are correctly classified, corresponding to a precision rate of 99.90%. Similarly, 23916 of 24275 microsphere images are accurately categorized, yielding a precision of 98.52%. Figure 4(d) showcases the distribution of cell size at 1 µm intervals as classified by the digitizer within the manual classification dataset, while the bottom part of Fig. 4(e) displays the distribution as 0.5 µm intervals.

The instability of cells focusing within the microfluidic channel occasionally results in the capture of out-of-focus images. Since our cell-size labeling method depends on signal amplitude, out-of-focus cells often produce weaker signals that can be masked by noise (shown in Fig. S4), leading to labeling inaccuracies. This issue is particularly pronounced in larger, out-of-focus cells, where weaker signals are more prevalent along the cell edges. Squamous epithelial cells, characterized by their flat, thin, and irregular shapes resembling fish scales^41^, are especially prone to such signal degradation, which may explain the lower labeling precision for larger cells than for smaller ones. The images acquired are based solely on intensity, providing only 2D information. In future iterations, integrating OTS quantitative phase imaging (OTS-QPI) could enable depth information capture, improving classification accuracy^43^.

### Rare events detected by the real-time cytometry in whole blood cell testing

Figure 4(f) presents some rare events discovered by our real-time optofluidic time-stretch imaging flow cytometry in a single whole blood sample test. As shown in Fig. 4f, in addition to common blood cell types such as erythrocytes, granulocytes, lymphocytes, and single platelets, we have also discovered some rare events, such as cell aggregation, platelet aggregation (which will lead to thrombotic disorders)^24^, monocytes, and the phenomenon of intercellular compression deformation and cell rupture caused by high-speed flow in microchannel. Detecting these rare phenomena becomes feasible through a single blood cell test using our advanced cytometry technology in real-time, which is facilitated by our cytometry’s continuous data stream transfer and real-time analysis capabilities, an unattainable advantage through data acquisition with an oscilloscope. This proof-of-concept demonstration showcases the potential application scenarios of our real-time cytometry, such as high-throughput cell deformability measurements^44,45^, rare cell detection in cancer screening^26,46^, and more.

### Typing of tumors and normal tissues in the colorectum

Over the past decade, the incidence of colorectal cancer has been rising in individuals under the age of 50, with global projections estimating 1.2 million cases by 2040^47–49^. To integrate our system into clinical applications, we trained a convolutional neural network (CNN) model to classify colorectal tumors and normal tissues. Given the wide variety of cell types in clinical colorectal samples, isolating individual cells with high purity and yield poses a significant challenge. To overcome this, we adopted a group-based classification approach by randomly grouping the images of each sample as 64 images as a group, instead of classifying each image individually. This method transforms the task into determining whether these grouped images collectively exhibit features indicative of the tumor. This allows us to determine if the grouped images exhibit features relevant to the tumor. To improve classification reliability, we incorporate an ensemble learning-inspired voting mechanism, where each image group is analyzed for cancer-specific features, and the final classification is based on a majority vote across all groups. This method has been demonstrated in the typing of leukemia before in our previous work^50^.

**Fig. 5:**
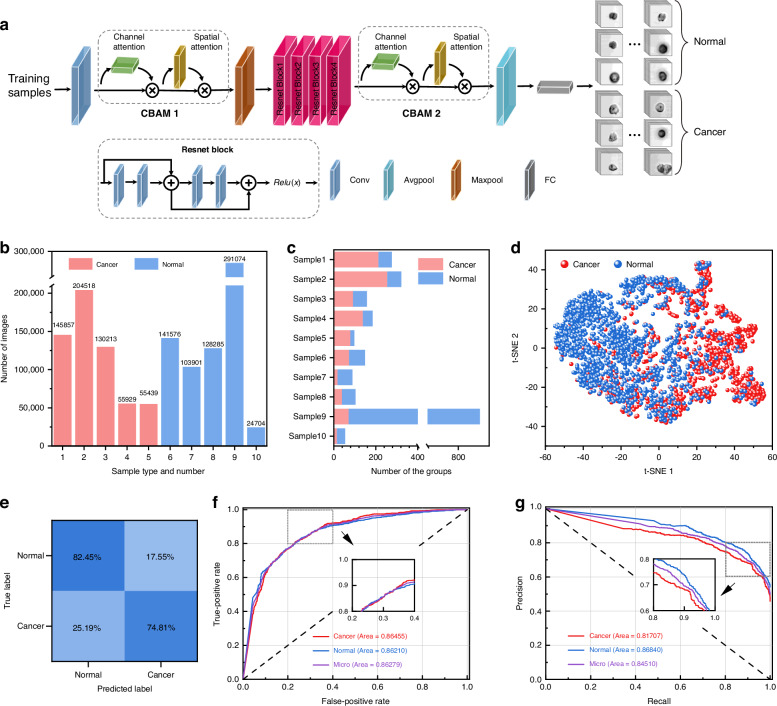
Performance of our proposed OTS-IFC in colorectal cancer typing. **a** The CNN architecture employed for colorectal cancer typing. **b** Total number of images acquired from the 10 clinical samples. **c** Heatmap showing the classification results of image groups across all 10 samples, along with the number of image groups per sample. **d** t-SNE plot depicting the separation of image groups between colorectal tumor and normal tissue samples. **e** Confusion matrix of the colorectal cancer typing. **f**, **g** ROC and PR curves of the typing of colorectal tumor and normal tissue

The multiple channels CNN structure, illustrated in Fig. 5(a), leverages ResNet18 for efficient feature extraction. Given the uneven distribution of disease-related cells within the groups, we incorporate a convolutional block attention module (CBAM) before and after ResNet18. This enables the network to focus on critical features while filtering out irrelevant ones. Figure 5(b) displays a histogram of the total number of acquired images per sample, with red bars representing colorectal tumor and blue bars representing normal tissue, a total of 1,281,496 cell images were acquired from 10 samples. These images were gated and threshold-filtered based on cell size labeled by FPGA. After eliminating impurities such as non-cell particles, cell debris, and small artifacts commonly found in clinical samples, the remaining images were split into training and test datasets with a 3:2 ratio. Further details on the samples and model training process are provided in the “Materials and Methods” section and Fig. S5.

To provide an intuitive and visual understanding of our CNN’s performance in colorectal tumor typing, quantitative analyses are presented in Fig. 5(c)-(g). Figure 5(c) illustrates a histogram showing the total number of groups for each sample, and different colors in histogram the indicate the number of groups identified as specific tissue types. Samples 1-5 show the majority of groups voting for colorectal tumors, while samples 6-10 predominantly vote for normal tissue, all aligning with clinical diagnoses. Figure 5(d) shows a t-distributed stochastic neighbor embedding (t-SNE) plot, where each point represents a 64-image group, with different colors denoting distinct tissue types. The convergence of image groups within the same sample highlights the method’s ability to differentiate sample types accurately. In Fig. 5(e), a confusion matrix displays the classification accuracy of all groups, with an accuracy of up to 82.45% for normal tissue types. Although some groups are misclassified, almost all samples are correctly identified, demonstrating the robustness and effectiveness of our voting-based CNN. Further, Fig. 5(f) shows the receiver operating characteristic (ROC) curves, with area under the curve (AUC) values of 0.8645 for colorectal tumors and 0.8661 for normal tissue, yielding a micro-average AUC above 0.8628. Precision-recall (PR) curves in Fig. 5(g) reveal AUC values of 0.8171 for colorectal tumors and 0.8684 for normal tissue, with a micro-average exceeding 0.8451. These results—AUC values consistently above 0.81 and micro-averages exceeding 0.84—underscore the accuracy and efficacy of our CNN model in colorectal tumor classification.

## Discussion

### The effect of motion blur on our cytometer

The OTS technology used in our system is designed to eliminate motion blur, even when imaging cells at high velocities. OTS stretches the optical signal temporally before digitization, allowing us to capture detailed images of fast-moving cells with high temporal resolution. However, to minimize photodamage on cells and reduce spectral distortion of pulses, we position the temporal disperser before the first spatial disperser in our OTS imaging system, which will increase motion blur. This configuration extends the exposure time per pulse from the femtosecond scale to 5.5 ns, leading to more significant relative spatial displacement of the pulse’s head and tail as they interact with the flowing cells, thus contributing to motion blur. As flow velocity increases from 1 m/s to 15 m/s, the theoretical relative displacement per pixel ranges from 5.5 nm to 82.5 nm. At 15 m/s, this displacement represents approximately 10.58% of the system’s theoretical resolution of 780 nm. Despite this, the motion blur remains within acceptable limits. Theoretically, placing the temporal disperser after the second spatial disperser could further alleviate motion blur and improve image quality at ultra-high flow velocities.

### The selection of FOV in the OTS-IFC

The selection of FOV needs to align with the design of the microfluidic chip. Theoretically, as the channel width of the chip decreases, the required FOV and sampling rate also decrease. However, when the width of the channel is close to the size of the cell, it is very easy to cause cell deformation. Therefore, the channel width is usually chosen to be 3 to 5 times larger than the cell size. Even with a highly efficient focusing system that keeps cells within a small observation window, there will still be cases where cells move outside the focused region. While a larger FOV could capture these rare events, it would also increase data redundancy. A more efficient approach involves event triggering on the fast axis, but due to limitations in ADC sampling rates and FPGA operating frequencies, our current system only triggers events on the slow axis (scan direction). Although fast-axis triggering is technically feasible, it requires higher sampling rates and faster FPGAs. In practical applications, selecting the FOV based on the cell size of the target sample while taking into account the focusing efficiency of the microfluidic channel is more effective, especially when the channel’s focusing is highly efficient.

### Further consideration about our cytometer integrated with other clinical applications

In clinical samples, the cellular composition is highly complex, and the detection of specific events or cell types is often a minority occurrence, particularly in applications such as cancer detection. To address these challenges, integrating OTS-IFC with artificial intelligence (AI) and machine learning (ML) is essential. In this study, we present a label-free method for classifying colorectal tumors and normal tissue, particularly suited for scenarios with no labels and involving high sample complexity. Although a more rigorous approach would involve manual annotation to construct a training dataset, there are two feasible methods for achieving this: acquiring images from high-purity samples or using fluorescence markers to selectively trigger the acquisition of target cells.

The complete blood count (CBC) serves as an example of a clinical application ideally suited for OTS-IFC, given that it naturally provides high-concentration samples, which align with the system’s detection throughput^51^. High-purity samples, such as those from leukemoid reactions, red blood cell suspensions, or apheresis platelets, can be used to capture single-cell images of white blood cells, red blood cells, and platelets, respectively. In subsequent five-part leukocyte classification, lymphocytes, granulocytes, and monocytes can be distinguished by size, while further differentiation of neutrophils, eosinophils, and basophils can be achieved using fluorescence markers to selectively trigger the acquisition of these specific cell types. Once a comprehensive database of blood cells is established, OTS-IFC has the potential to generate more rapid and precise diagnostic reports than traditional CBCs.

Currently, numerous studies have verified the potential application of OTS-IFC in blood sample analysis within clinical settings^24,50,52,53^. As AI and ML technologies continue to evolve, integrating the immense data acquisition capabilities of our cytometry system with big data models will be pivotal in enabling precise disease prediction and diagnosis. This advancement will facilitate early detection and personalized treatment for a wide range of diseases, positioning OTS-IFC as a critical tool in modern healthcare.

### Considerations on further updates and upgrades to our system

In this work, we present a redundant data removal scheme tailored to the characteristics of OTS imaging signals, enabling continuous data stream transfer to the host and real-time cell image reconstruction for general-purpose analytical applications. The parameterizable design of the algorithm allows it to be adapted easily to other OTS-IFCs. However, complex real-time biological and clinical analysis tasks require more sophisticated algorithms or ML models. For example, in this study, we employ CNNs to classify colorectal tumors and normal tissues, a task not yet implemented in real-time due to current hardware constraints. Currently, the FPGA design leverages a single-clock, multi-pixel input clock domain. While this approach improves throughput, it is less flexible than traditional single-clock, single-pixel designs. Although the multi-pixel configuration enhances processing speed, it restricts system adaptability, particularly when compared to software-based solutions, which are more flexible and easier to update. This limits the system’s ability to quickly accommodate new algorithms, a crucial requirement for dynamic, real-time applications.

To extend this capability, offloading data through the bus to a more flexible platform, such as a GPU or a data center accelerator card with FPGA logic resources, presents a promising solution for future system development. These platforms can be integrated with our cytometry setup on the same host, providing greater flexibility for algorithm deployment. With the rapid advancements in high-level synthesis (HLS) and associated toolchains^14,54–56^, we anticipate that complex algorithms and AI models can be deployed on our platform in significantly shorter timeframes by utilizing existing algorithm libraries. Additionally, structured sparse pruning and dynamic low-bit quantization technology can be employed to optimize and accelerate AI models^57^, which could decrease the demand for computing power and bandwidth requirements, thus reducing the complexity and cost of the system. These advancements will accelerate the adoption of OTS-IFC, enabling it to expand its applications to a broader array of real-time, high-throughput tasks.

### Comparison with the existing high-throughput imaging flow cytometry technologies

Table 1 presents a comparison of various high-throughput IFC techniques, including conventional IFC^4,58^, optical time-stretch (OTS)^25,26,43^, frequency-division multiplexing (FDM)^59–61^, virtual-freezing fluorescence imaging (VIFFI)^62,63^, temporal-spatial transformation^64^, and linear array spot excitation (LASE)^65^. In terms of imaging capabilities, conventional IFC and OTS both provide quantitative phase imaging, which could provide essential insights into the local thickness and refractive index of cellular structures. This capability is absent in the other methods listed. However, fluorescence imaging is available in most methods except OTS, making OTS less suitable for applications requiring this imaging modality, such as fluorescence-based biological assays.

**Table 1:** Comparison of the high-throughput imaging flow cytometry techniques

	Conventional IFC	OTS	FDM	VIFFI	Temporal-spatial transformation	LASE
Illumination	Laser, LED	Broadband pulse laser	CW laser	CW laser	CW laser, LED	CW laser
Throughput (eps)	1000	1,000,000	50,000	10,000	1000	10,000
Spatial resolution	High	High	High	High	Low	Moderate
Fluorescence imaging capability	Yes	No	Yes	Yes	Yes	Yes
Quantitative phase imaging capability	Yes	Yes	No	No	No	No
Detector	CMOS, CCD	PD	PMT, APD	sCMOS	PMT	PMT
Continuous transfer and storage at max throughput	Yes	Yes	Yes	No	No	No
Real-time imaging	Yes	Yes	Yes	Yes	No	No
Realization degree of real-time analysis of image	High	Moderate	Moderate	Low	No discussion	No discussion
References	^4,58^	^25,26,43^	^59–61^	^62,63^	^64^	^65^

*OTS* optical time-stretch, *FDM* frequency-division multiplexing, *VIFFI* virtual-freezing fluorescence imaging, *LASE* linear array spot excitation, *CW* continuous wave

Regarding corresponding data processing capabilities development, OTS, FDM, temporal-spatial transformation, and the LASE all require sophisticated algorithms to handle high-speed imaging. OTS and FDM have advanced support due to their early introduction and widespread use, enabling real-time imaging capabilities. OTS stands out with a remarkable throughput of 1,000,000 eps, positioning it as the only technique comparable to conventional flow cytometry (with a throughput of ~100,000 eps), making it particularly well-suited for analyzing large-volume and high-concentration samples, such as whole-blood or environmental samples.

Another key distinction is OTS’s massive raw data generation, which reaches gigapixels per second or even higher, far exceeding other techniques. This immense data throughput places substantial demands on hardware, algorithm design, and computational power. The high throughput necessitates advanced solutions for efficient data flow management to maintain real-time processing capabilities. This paper proposes strategies to address these challenges and outlines methods for future upgrades, ensuring the continued advancement of real-time imaging flow cytometry applications.

### Summary

In summary, this paper presents a redundant data removal scheme specifically designed for OTS imaging signals, and we provide both theoretical analysis and experimental validation. This enables continuous data stream transfer to the host and real-time cell image reconstruction. By co-designing the optical imaging system, a 3D-focusing microfluidic chip, and a data acquisition and processing system equipped with an online ADC and FPGA, we achieved a record-breaking detection throughput of 1,000,000 eps, with an exceptional spatial resolution of 780 nm—the highest performance reported globally. This represents an improvement of 2 to 3 orders of magnitude over existing technologies, making theoretical performance indices a practical reality. This technical breakthrough offers new perspectives and opens possibilities for imaging flow cytometry.

Additionally, our proposed cytometer demonstrates remarkable accuracy in real-time cell classification, the ability to detect rare events in whole blood samples, and effective integration with clinical applications for classifying colorectal tumors and normal tissues. These experiments highlight the effectiveness and adaptability of our proposed method. Finally, we discuss future directions for further integrating OTS-IFC into clinical applications and updating and iterating the system, particularly in enhancing the capability for real-time processing and analysis of large datasets. We believe that this work provides valuable insights into the field of imaging flow cytometry and promotes the widespread application of OTS-IFC in life sciences and biomedical research.

## Materials and methods

### Architecture of the sampling and processing device

**Fig. 6:**
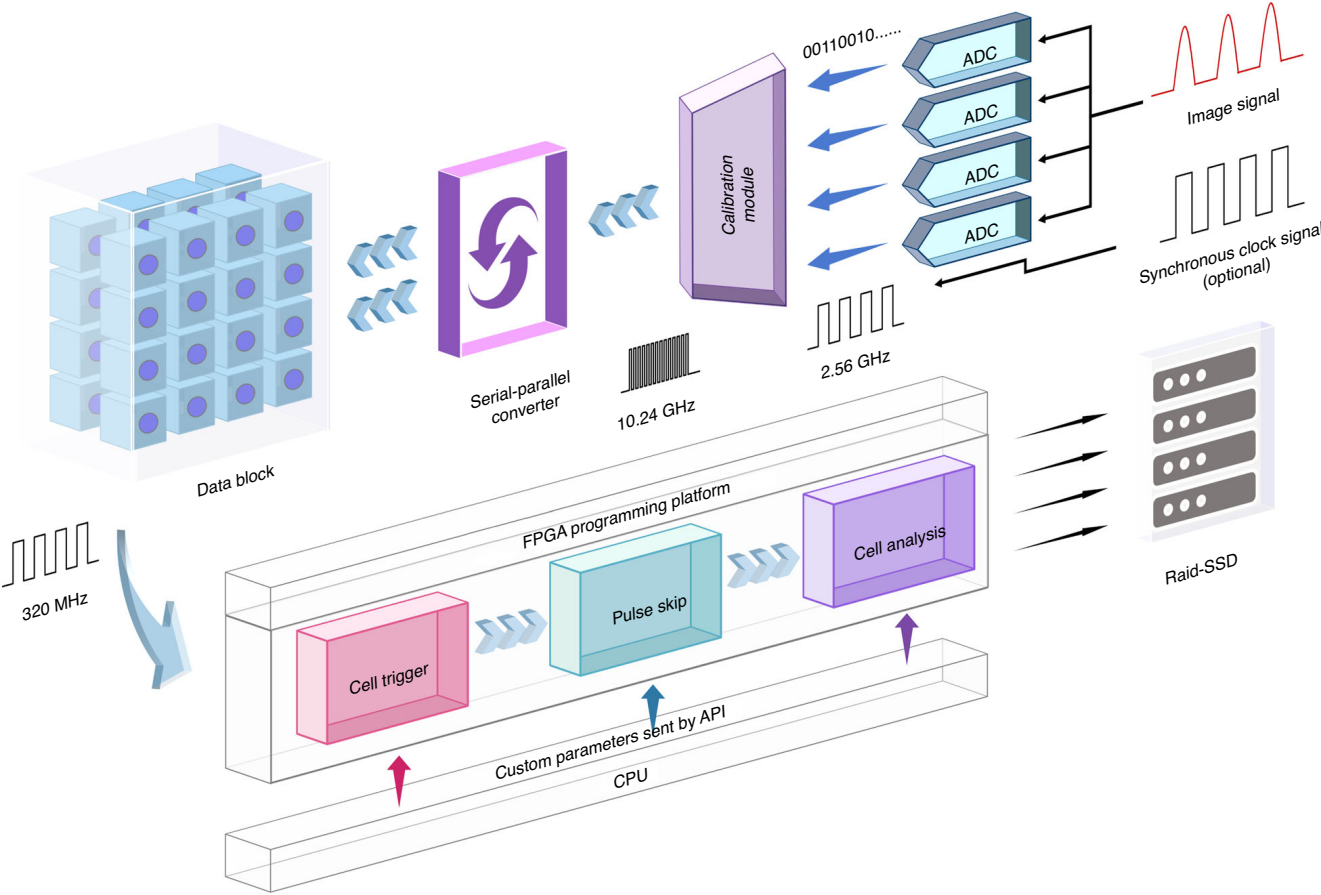
The data acquisition and processing flow of our optofluidic time-stretch imaging flow cytometry

The structure of the high-speed sampling and processing device is shown in Fig. 6. Four ADCs, synchronized with the pulse laser and working at a sampling frequency of 2.56 GHz, are configured as temporally interleaved to achieve an equivalent sampling rate of 10.24 GS/s. The data is first adjusted and compensated by the calibration module and then sent to the FPGA after being transformed into 32 parallel streams. Therefore, the FPGA can process the high-speed data stream in parallel at the clock frequency of 320 MHz. The ADC is configured to collect the data with a sampling rate of ~10 GS/s and a quantization bit number of 16, meaning that the ADC generates ~20 GB/s raw data for the FPGA, which is far beyond the capability of the PCIe ×8 Gen3 interface connecting the FPGA and the host PC. So, to make sure the image data of the cells can be received without loss, FPGA has to reduce the redundancy of the data and shrink the data stream below 5.6 GB/s, which is the stable continuous data transmission rate of the PCIe ×8 Gen3 interface.

### The principle of event trigger and redundancy removal in our OTS-IFC

As shown in Fig. S2, much redundant data will be generated by optofluidic time-stretch imaging, so we remove redundancy in the original image data by deploying algorithms on FPGA to meet sustained data transfer and reduce repeated handle of redundant data. We recapitulate the redundant data that can be discarded in the image into four parts and briefly discuss the data reduction proportion (DRP) realized after deploying algorithms to remove the redundant data in each part (More quantitative analyses are available in Supplementary S2). First of all, the noise signal between adjacent pulses does not carry any image information, which is redundant data that can be discarded, and the pulse duty ratio determines the DRP. Secondly, we are only concerned about the region of interest (ROI) that includes cells, and the data between cell and cell carry useless information, so the pulse betwixt frames is superfluous data that can be discarded. The main principle of ROI detection is described in Fig. S2 (i.e., cell trigger principle). The predominant difference between the pulse that passes through a cell and that does not is signal amplitude. Hence, FPGA calculates the average of each pulse (AOP) to engender the AOP signal in Fig. S2 and then sets an appropriate threshold to trigger each frame of the cell image. Obviously, the actual throughput of cells decides the DRP. Removing the two parts’ redundancy on FPGA is shown in step 1 of Fig. S2, which corresponds to cropping the cell image. Thirdly, although the pulse signal is 16-bit quantized by ADC, 16-bit quantization of the image data is unnecessary, 8-bit data is sufficient to show the image’s detail after removing the background on FPGA, and the DRP is plainly given as 1/2.

Finally, when cells do not reach the theoretical upper limit of flowing rate in the microfluidic channel (depending on the design scheme of OTS-IFC), two or even dozens of adjoining pulses convey identical image 1D (line) information, which is tautological data that can be discarded. Assuming that one sample point represents the theoretical spatial resolution of our optofluidic time-stretch microscopy of around 0.8 µm, the cell image with a field of view (FOV) of 30 um in flow direction must contain at least 38 pulses, equal to 30 µm FOV cell image acquires by our cytometry at the limit flowing rate of 64 m/s. As illustrated in step 2 of Fig. S2, a cell image with a field of view (FOV) of 30 µm acquired at 1 m/s contains 2400 pulses, so FPGA acts different pulse skip factors to the cell image to reduce the pulses contained in each image, which could further reduce the DR. For example, removing seven pulses of eight consecutive pulses is equivalent to acting the skip factor of 8 to the cell image, and the number of pulses reduces from 2400 to 300 in each cell image. The maximum DRP is determined by the practical flowing rate and theoretical maximum flowing rate of cells, and the theoretical maximum flowing rate of our cytometry is 64 m/s, which meets the condition that contains adequate pixels in the flow direction to ensure image quality without considering cells and microfluidic channel tolerance to the hydrodynamic pressure. Depending on the experiment’s situation, a total DRP of more than 18/25 must be applied after development for the continuous data transfer (i.e., the DR is less than 5.6 GB/s).

### Cell image reconstruction and analysis algorithm on FPGA

**Fig. 7:**
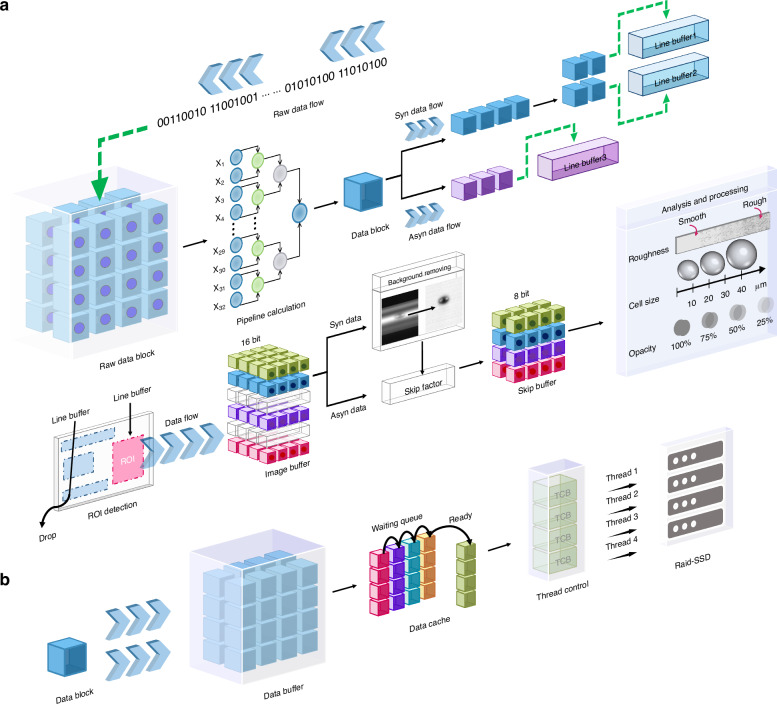
Real-time cell image formation, analysis, and data storage principle of our optofluidic time-stretch imaging flow cytometry. **a** The procedure of real-time cell image formation and analysis. In a comprehensive FPGA-based pipeline, cell images are initially prepared through data packing and real-time line buffer formation based on synchronous or asynchronous configurations, followed by an ROI detection step, image background removal, and morphological analysis, and the FPGA eventually adding critical data for decision-making by CPU. **b** The procedure of real-time cell image data storage. Our approach employs the producer-consumer model to transfer data and maintain uniformity for easy analysis

To achieve high-throughput cell imaging and analysis, we design and deploy the high-performance algorithm in the FPGA to conduct online data processing in real time. As depicted in Fig. 7(a), the cell image reconstruction, redundancy elimination, and analysis algorithm are deployed on the FPGA in the form of a pipeline. This setup is meticulously designed to accommodate sustained data transfer and real-time processing of cell images. Firstly, the data is packed into a data block containing 32 sample points for the FPGA real-time parallel process to make the balance of throughput. Subsequently, to make the timing closure of the whole design, the FPGA calculates the data block average using the two-divide way to obtain the calculation result for subsequent use. The formation of the line buffer in the data flow depends on the synchronous or asynchronous configuration. In the synchronous configuration data flow, the line buffer is created every 4 cycles and is subsequently split into 2 data blocks. This division is based on a pulse threshold value set by the host, which exactly covers the pulse width. In the asynchronous configuration data flow, due to the non-integer-period-sampling effect, the line buffer needs to use 3 data blocks based on the pulse threshold value to encompass pulse width adequately. Secondly, once a line buffer is formed, the ROI detection step starts, also known as the cell trigger step. This detection principle is based on the frame temporal difference, with a specific ROI threshold determining which line buffer should be cached in the image buffer. Simultaneously, for data flow of the synchronous configuration, the image line background will be removed from the image buffer, and then the 16-bit data is re-quantized to the 8-bit data (sufficient to describe the image’s detail) to reduce redundancy further^25,26,35,36,66,67^. Thirdly, depending on the current cell flow rate of the system, oversampled pulses in the flow direction are removed by using the corresponding skip factor, and the imaging buffer will transform into the skip buffer. Finally, the image analysis processing is launched, and kinds of morphological information, such as cell size, granularity, and opacity, are obtained from each detection cell^66–69^.

The image analysis algorithms are implemented on the analysis module with 32-pixel parallel input per clock cycle, which differs from the traditional single-clock, single-pixel designs to maintain the balanced data throughput. Once the image is formed, a binary mask is generated through binarization. For a binary image $B(x.y)$, the cell area $A$ (in pixels) is computed as follows:

1 $$A=\mathop{\sum }\limits_{x=1}^{M}\mathop{\sum }\limits_{y=1}^{N}B(x,y)$$

cells are then labeled based on their size by calculating the equivalent circular diameter of the cell’s area.

Since the OTS intensity image reflects the transmittance of the cell, the opacity of the cell can be quantified by calculating the total intensity value of image pixels within the mask. It can be expressed as:

2 $${I}_{{total}}=\mathop{\sum }\limits_{x=1}^{M}\mathop{\sum }\limits_{y=1}^{N}I(x,y)\cdot B(x,y)$$

Additionally, based on the computational constraints under this clock domain, we also used the following way to define the granularity G of the cell using the following formula:

3 $$G=k\cdot ({N}_{x}+{N}_{y})$$

where $k$ is a constant of proportionality that adjusts the scale of granularity, and ${N}_{x}$ and ${N}_{y}$ is the number of inflection points along the mean curve in the x-axis and y-axis separately. Furthermore, FPGA appends this information into the data header of each cell frame data for subsequent individual decision-making by the CPU based on various application purposes.

### Data caching architecture for continuous image data storage

After discarding the redundancy data on FPGA, image data is transferred to the PC through the PCI-E interface and then stored on SSD for subsequent analysis, but directly writing single-segment cell image data to the SSD can not meet continuous image data storage under such a large DR. To address this challenge, we devised a unique data caching architecture tailored to accommodate the continuous image data store. As illustrated in Fig. 7(b), our approach adopts a producer thread to fetch a data buffer containing multi-segment cell image data from the digitizer. Once the data buffer is sent to the memory pool, awaiting writing, the producer thread returns to continue fetching the data buffer. Multiple consumer threads are employed to write the queued data buffers from the memory pool to the RAID-SSD in parallel, following the first-in, first-out (FIFO) principle. The consolidation of multi-segment cell image data into a data buffer leads to a significant reduction in both data fetching and writing operations. Additionally, the data write speed of RAID-SSD is beyond the throughput of PCIe x8 Gen3 in theory. These two combined factors ensure uninterrupted image data storage. Furthermore, the FPGA establishes fixed parameters for the number of pulses within each segment of image data and the time window used to capture these pulses. This parameter uniformity ensures a constant length for single-segment cell image data, simplifying its separation from the data buffer for subsequent analysis. The method performance is reported in our previous work^70^.

### CNN model training details

Samples 1–5 were obtained from tumors of colorectal cancer patients, while samples 6–10 were collected from normal colorectal tissues of the same patients. A total of 1,281,496 cell images were acquired from these 10 samples at a flow rate of 5 m/s, using a data acquisition platform consisting of an Intel Core i5-12600K CPU @ 3.70 GHz, 16GB of Kingston DDR5 5200 MHz RAM, a RAID 0 disk array formed by two Samsung PM9A1 SSDs, and a Teledyne SP Devices ADQ7DC digitizer. The cell size distribution for these samples, calculated by the FPGA (Xilinx Kintex UltraScale xcku085-fiva1517-2-e), is shown in Fig. S5(a). The images were subsequently gated and threshold-filtered using a 10 µm size threshold to exclude particles with a high probability of irrelevance. This process yielded 167,037 images from tumor samples (28.22%) and 196,896 images from normal tissue samples (28.56%). Impurities such as non-cell particles, cell debris, and small artifacts, which are common in clinical samples, were removed during the filtering process. Fig. S5(b) presents the image library of tumor and normal tissues after the threshold gating. The remaining images were split into training and test datasets in a 3:2 ratio. The ML model was then trained on a separate server running Pytorch on an Ubuntu platform equipped with an Intel Xeon Platinum 8352S @ 2.20 GHz CPU, 32GB of DDR4 3200 MHz RAM, and an NVIDIA A6000 GPU with 48GB memory. Detailed information on the CNN architecture is provided in Table S2.

### Fabrication of the microfluidic device

We developed a high-intensity microfluidic chip optimized for high-speed and high-throughput cell detection. The 3D focusing structure enhances the probability of cells being focused, thereby improving the event rate. As the flow rate in the channel increases, the probability of cells being focused in the center of the channel (FOV) and the imaging focal plane increases, thereby reducing the likelihood of cell overlap. The microfluidic device features a sandwich-like structure, with two pieces of slide glasses bonding on the two surfaces of the main PDMS chip, which contains the microchannel. The connections of the microchannel and pipeline are inserted into PDMS horizontally to minimize the structure and avoid interference with the objectives. This kind of structure allows an extremely high flow rate and velocity in the microchannel.

The microchannel is fabricated using standard soft lithography methods. The negative photoresist of Su8 (MicroChem Corp., Newton, MA) was used to fabricate the mold, and mixed PDMS (SYLGARD 184, Dow Corning, Midland, MI, USA) was poured over the mold and heated under 80 °C for six hours for solidification. The horizontal holes are first punched by a puncher from the PDMS chip’s side, and then the vertical holes are punched to connect the horizontal holes and microchannel to realize the horizontal connection. The residual PDMS of the horizontal hole can be picked out after the vertical hole is punched. As all holes are fabricated, two pieces of slide glasses are bonded on the two surfaces of the PDMS chip after two turns of plasma treatments. The stainless-steel capillary tubes are inserted into the horizontal holes as the connections. At last, Araldite standard epoxy (Huntsman Corp, Basel, Switzerland) was used to fill the interval between the two glasses, sealing the PDMS chip and the connections. The microchannel performance is reported in our previous work^71^.

### Sample preparation

The blood samples utilized in this study were acquired through a conventional venous blood collection procedure from healthy donors. Subsequently, the collected whole blood was temporarily preserved in test tubes containing an ethylenediaminetetraacetic acid (EDTA) anticoagulant to prevent clotting. Following this, the samples were systematically diluted into varying concentrations using a sodium chloride solution. The 15 µm polystyrene microspheres (ACME FG15UM-10, 15 µm) were mixed with a diluted blood sample to ensure that the concentration of the two was as consistent as possible to perform one of the particle classification experiments.

Cervical cell samples in this study were obtained from clinical sampling. Specifically, a sample is obtained from the cervix of a donor by gently rotating the brush of a cervical sampler several times. Subsequently, the obtained brush was soaked in a bottle containing the cell preservation solution (ThinPrep, PreseruCyt Solution) so that the cells could dissolve in the bottle.

The colorectum cell samples used in this study were obtained from specimens following radical resection of colorectal cancer, after dissection of the primary specimen post-surgery. Specifically, samples were collected by gently rotating a sampling brush over both the tumor and normal intestinal mucosa several times. The cells collected on the brush were then submerged in a bottle containing cell preservation solution (ThinPrep, PreservCyt Solution) to ensure fixation and preservation. These samples were subsequently used for imaging flow cytometry analysis.

This study was approved by the Medical Ethics Committee of Zhongnan Hospital of Wuhan University, the Medical Ethics Committee of People’s Hospital of Anshun, and the Medical Ethics Committee of Peking University First Hospital. All experiments were performed in accordance with relevant laws, guidelines, and regulations. Written informed consents were obtained from the donors.

## Supplementary information

Supplementary Information for Imaging flow cytometry with a real-time throughput beyond 1,000,000 events per second

Workflow of the imaging flow cytometer

Comparison of online and offline image acquisition

Performance of redundant data removal in our system

## Data Availability

Data may be obtained upon reasonable request.

## References

[1] Nitta, N. et al. Intelligent image-activated cell sorting. *Cell* **175**, 266–276.e13 (2018). 30166209 10.1016/j.cell.2018.08.028

[2] George, T. C. et al. Distinguishing modes of cell death using the ImageStream® multispectral imaging flow cytometer. *Cytom. Part A* **59A**, 237–245 (2004). 10.1002/cyto.a.20048 15170603

[3] Litvinenko, A. L. et al. Fluorescence-free flow cytometry for measurement of shape index distribution of resting, partially activated, and fully activated platelets. *Cytom. Part A* **89A**, 1010–1016 (2016). 10.1002/cyto.a.23003 27768824

[4] Basiji, D. A. et al. Cellular image analysis and imaging by flow cytometry. *Clin. Lab. Med.* **27**, 653–670 (2007). 17658411 10.1016/j.cll.2007.05.008 PMC2034394

[5] Song, P. M. et al. Optofluidic ptychography on a chip. *Lab a Chip* **21**, 4549–4556 (2021). 10.1039/d1lc00719j 34726219

[6] Huang, K. R. et al. Deep imaging flow cytometry. *Lab a Chip* **22**, 876–889 (2022). 10.1039/d1lc01043c 35142325

[7] Maryanovich, M. et al. An MTCH2 pathway repressing mitochondria metabolism regulates haematopoietic stem cell fate. *Nat. Commun.* **6**, 7901 (2015). 26219591 10.1038/ncomms8901

[8] Porichis, F. et al. High-throughput detection of miRNAs and gene-specific mRNA at the single-cell level by flow cytometry. *Nat. Commun.* **5**, 5641 (2014). 25472703 10.1038/ncomms6641 PMC4256720

[9] Sancho, D. et al. Identification of a dendritic cell receptor that couples sensing of necrosis to immunity. *Nature* **458**, 899–903 (2009). 19219027 10.1038/nature07750 PMC2671489

[10] Blasi, T. et al. Label-free cell cycle analysis for high-throughput imaging flow cytometry. *Nat. Commun.* **7**, 10256 (2016). 26739115 10.1038/ncomms10256 PMC4729834

[11] Tiwari, V., Sutton, M. A. & McNeill, S. R. Assessment of high speed imaging systems for 2D and 3D deformation measurements: methodology development and validation. *Exp. Mech.* **47**, 561–579 (2007).

[12] Nishino, N. et al. High-speed 2-D image measurement for plasma-wall interaction studies. *J. Nucl. Mater.* **337-339**, 1073–1076 (2005).

[13] Thoroddsen, S. T., Etoh, T. G. & Takehara, K. High-speed imaging of drops and bubbles. *Annu. Rev. Fluid Mech.* **40**, 257–285 (2008).

[14] Lee, D. et al. A hardware accelerated system for high throughput cellular image analysis. *J. Parallel Distrib. Comput.* **113**, 167–178 (2018).

[15] Urbanska, M. et al. A comparison of microfluidic methods for high-throughput cell deformability measurements. *Nat. Methods* **17**, 587–593 (2020). 32341544 10.1038/s41592-020-0818-8 PMC7275893

[16] Xing, H. Z. et al. High-speed photography and digital optical measurement techniques for geomaterials: fundamentals and applications. *Rock. Mech. Rock. Eng.* **50**, 1611–1659 (2017).

[17] Zlatanski, M., Uhring, W. & Le Normand, J. P. Sub-500-ps temporal resolution streak-mode optical sensor. *IEEE Sens. J.* **15**, 6570–6583 (2015).

[18] Etoh, T. G. et al. The theoretical highest frame rate of silicon image sensors. *Sensors* **17**, 483 (2017). 28264527 10.3390/s17030483 PMC5375769

[19] Goda, K., Tsia, K. K. & Jalali, B. Amplified dispersive Fourier-transform imaging for ultrafast displacement sensing and barcode reading. *Appl. Phys. Lett.* **93**, 131109 (2008).

[20] Goda, K., Tsia, K. K. & Jalali, B. Serial time-encoded amplified imaging for real-time observation of fast dynamic phenomena. *Nature* **458**, 1145–1149 (2009). 19407796 10.1038/nature07980

[21] Goda, K. et al. High-throughput optical coherence tomography at 800 nm. *Opt. Express* **20**, 19612–19617 (2012). 23037013 10.1364/OE.20.019612

[22] Guo, Q. et al. High-speed compressive microscopy of flowing cells using sinusoidal illumination patterns. *IEEE Photonics J.* **9**, 3900111 (2017).

[23] Wu, J. L. et al. Multi-MHz laser-scanning single-cell fluorescence microscopy by spatiotemporally encoded virtual source array. *Biomed. Opt. Express* **8**, 4160–4171 (2017). 28966855 10.1364/BOE.8.004160 PMC5611931

[24] Jiang, Y. Y. et al. Label-free detection of aggregated platelets in blood by machine-learning-aided optofluidic time-stretch microscopy. *Lab a Chip* **17**, 2426–2434 (2017). 10.1039/c7lc00396j 28627575

[25] Lei, C. et al. High-throughput imaging flow cytometry by optofluidic time-stretch microscopy. *Nat. Protoc.* **13**, 1603–1631 (2018). 29976951 10.1038/s41596-018-0008-7

[26] Goda, K. et al. High-throughput single-microparticle imaging flow analyzer. *Proc. Natl Acad. Sci. USA* **109**, 11630–11635 (2012). 22753513 10.1073/pnas.1204718109 PMC3406874

[27] Chen, C. L. et al. Deep learning in label-free cell classification. *Sci. Rep.* **6**, 21471 (2016). 26975219 10.1038/srep21471 PMC4791545

[28] Wu, Y. Z. et al. Intelligent frequency-shifted optofluidic time-stretch quantitative phase imaging. *Opt. Express* **28**, 519–532 (2020). 32118978 10.1364/OE.380679

[29] Wu, J. L. et al. Ultrafast laser-scanning time-stretch imaging at visible wavelengths. *Light Sci. Appl.* **6**, e16196 (2017). 30167195 10.1038/lsa.2016.196 PMC6061895

[30] Lai, Q. T. K. et al. High-speed laser-scanning biological microscopy using FACED. *Nat. Protoc.* **16**, 4227–4264 (2021). 34341580 10.1038/s41596-021-00576-4

[31] Lee, K. C. M. et al. Multi-ATOM: ultrahigh-throughput single-cell quantitative phase imaging with subcellular resolution. *J. Biophotonics* **12**, e201800479 (2019). 30719868 10.1002/jbio.201800479 PMC7065649

[32] Shi, R. B. et al. A real-time coprime line scan super-resolution system for ultra-fast microscopy. *IEEE Trans. Biomed. Circuits Syst.* **13**, 781–792 (2019). 31059454 10.1109/TBCAS.2019.2914946

[33] Chan, A. C. S. et al. All-passive pixel super-resolution of time-stretch imaging. *Sci. Rep.* **7**, 44608 (2017). 28303936 10.1038/srep44608 PMC5356014

[34] Goda, K. et al. Hybrid dispersion laser scanner. *Sci. Rep.* **2**, 445 (2012). 22685627 10.1038/srep00445 PMC3370333

[35] Mahjoubfar, A. et al. Time stretch and its applications. *Nat. Photonics* **11**, 341–351 (2017).

[36] Weng, Y. Y. et al. Analysis of signal detection configurations in optical time-stretch imaging. *Opt. Express* **28**, 29272–29284 (2020). 33114830 10.1364/OE.403454

[37] Liu, A. M., Lin, W. S. & Narwaria, M. Image quality assessment based on gradient similarity. *IEEE Trans. Image Process.* **21**, 1500–1512 (2012). 22106145 10.1109/TIP.2011.2175935

[38] Wang, Z. et al. Image quality assessment: from error visibility to structural similarity. *IEEE Trans. Image Process.* **13**, 600–612 (2004). 15376593 10.1109/tip.2003.819861

[39] Sheikh, H. R. & Bovik, A. C. Image information and visual quality. *IEEE Trans. Image Process.* **15**, 430–444 (2006). 16479813 10.1109/tip.2005.859378

[40] Pan, X. W. et al. MobileNet-light: a lightweight TCT image classification model for cervical cancer. Proceedings of the 2023 International Joint Conference on Neural Networks. Gold Coast, Australia: IEEE, 2023.

[41] Chen, H. et al. CytoBrain: cervical cancer screening system based on deep learning technology. *J. Computer Sci. Technol.* **36**, 347–360 (2021).

[42] Cohen, P. A. et al. Cervical cancer. *Lancet* **393**, 169–182 (2019). 30638582 10.1016/S0140-6736(18)32470-X

[43] Deng, Y. J. et al. Studying the efficacy of antiplatelet drugs on atherosclerosis by optofluidic imaging on a chip. *Lab a Chip* **23**, 410–420 (2023). 10.1039/d2lc00895e 36511820

[44] Otto, O. et al. Real-time deformability cytometry: on-the-fly cell mechanical phenotyping. *Nat. Methods* **12**, 199–202 (2015). 25643151 10.1038/nmeth.3281

[45] Fregin, B. et al. High-throughput single-cell rheology in complex samples by dynamic real-time deformability cytometry. *Nat. Commun.* **10**, 415 (2019). 30679420 10.1038/s41467-019-08370-3 PMC6346011

[46] Stott, S. L. et al. Isolation of circulating tumor cells using a microvortex-generating herringbone-chip. *Proc. Natl Acad. Sci. USA* **107**, 18392–18397 (2010). 20930119 10.1073/pnas.1012539107 PMC2972993

[47] Siegel, R. L. et al. Colorectal cancer statistics, 2023. *CA: A Cancer J. Clinicians* **73**, 233–254 (2023). 10.3322/caac.21772 36856579

[48] Xi, Y. & Xu, P. F. Global colorectal cancer burden in 2020 and projections to 2040. *Transl. Oncol.* **14**, 101174 (2021). 34243011 10.1016/j.tranon.2021.101174 PMC8273208

[49] Biller, L. H. & Schrag, D. Diagnosis and treatment of metastatic colorectal cancer: a review. *JAMA* **325**, 669–685 (2021). 33591350 10.1001/jama.2021.0106

[50] Weng, Y. Y. et al. Typing of acute leukemia by intelligent optical time-stretch imaging flow cytometry on a chip. *Lab a Chip* **23**, 1703–1712 (2023). 10.1039/d2lc01048h 36799214

[51] Valiathan, R., Ashman, M. & Asthana, D. Effects of ageing on the immune system: infants to elderly. *Scand. J. Immunol.* **83**, 255–266 (2016). 26808160 10.1111/sji.12413

[52] Zhou, Y. Q. et al. Intelligent platelet morphometry. *Trends Biotechnol.* **39**, 978–989 (2021). 33509656 10.1016/j.tibtech.2020.12.012

[53] Zhou, Y. Q. et al. Intelligent classification of platelet aggregates by agonist type. *eLife* **9**, e52938 (2020). 32393438 10.7554/eLife.52938 PMC7217700

[54] Li, Z. et al. A high-performance pixel-level fully pipelined hardware accelerator for neural networks. IEEE Transactions on Neural Networks and Learning Systems, 10.1109/TNNLS.2024.3423664 (2024). 10.1109/TNNLS.2024.3423664 38995709

[55] Winterstein, F., Bayliss, S. & Constantinides, G. A. High-level synthesis of dynamic data structures: a case study using vivado HLS. Proceedings of the 2013 International Conference on Field-Programmable Technology. Kyoto, Japan: IEEE, 2013, 362-365.

[56] de Fine Licht, J. et al. Transformations of high-level synthesis codes for high-performance computing. *IEEE Trans. Parallel Distrib. Syst.* **32**, 1014–1029 (2021).

[57] Han, S. et al. EIE: efficient inference engine on compressed deep neural network. Proceedings of 2016 ACM/IEEE 43rd Annual International Symposium on Computer Architecture. Seoul, Korea (South): IEEE, 2016, 243-254.

[58] Rees, P. et al. Imaging flow cytometry. *Nat. Rev. Methods Prim.* **2**, 86 (2022). 10.1038/s43586-022-00167-x PMC10468826 37655209

[59] Schraivogel, D. et al. High-speed fluorescence image-enabled cell sorting. *Science* **375**, 315–320 (2022). 35050652 10.1126/science.abj3013 PMC7613231

[60] Nishikawa, M. et al. Massive image-based single-cell profiling reveals high levels of circulating platelet aggregates in patients with COVID- 19. *Nat. Commun.* **12**, 7135 (2021). 34887400 10.1038/s41467-021-27378-2 PMC8660840

[61] Diebold, E. D. et al. Digitally synthesized beat frequency multiplexing for sub-millisecond fluorescence microscopy. *Nat. Photonics* **7**, 806–810 (2013).

[62] Matsumura, H. et al. Virtual-freezing fluorescence imaging flow cytometry with 5-aminolevulinic acid stimulation and antibody labeling for detecting all forms of circulating tumor cells. *Lab a Chip* **23**, 1561–1575 (2023). 10.1039/d2lc00856d 36648503

[63] Mikami, H. et al. Virtual-freezing fluorescence imaging flow cytometry. *Nat. Commun.* **11**, 1162 (2020). 32139684 10.1038/s41467-020-14929-2 PMC7058616

[64] Han, Y. Y. & Lo, Y. H. Imaging cells in flow cytometer using spatial-temporal transformation. *Sci. Rep.* **5**, 13267 (2015). 26281956 10.1038/srep13267 PMC4539609

[65] Han, Y. et al. Imaging flow cytometry using linear array spot excitation. *Device* **1**, 100124 (2023).

[66] Guo, B. S. et al. High-throughput, label-free, single-cell, microalgal lipid screening by machine-learning-equipped optofluidic time-stretch quantitative phase microscopy. *Cytom. Part A* **91**, 494–502 (2017). 10.1002/cyto.a.23084 28399328

[67] Lei, C. et al. High-throughput label-free image cytometry and image-based classification of live Euglena gracilis. *Biomed. Opt. Express* **7**, 2703–2708 (2016). 27446699 10.1364/BOE.7.002703 PMC4948623

[68] Guo, B. S. et al. High-throughput label-free screening of Euglena gracilis with optofluidic time-stretch quantitative phase microscopy. Proceedings of SPIE 10076, High-Speed Biomedical Imaging and Spectroscopy: Toward Big Data Instrumentation and Management II. San Francisco, California, United States: SPIE, 2017.

[69] Lei, C. et al. GHz optical time-stretch microscopy by compressive sensing. *IEEE Photonics J.* **9**, 3900308 (2017).

[70] Hou, D. et al. Optofluidic time-stretch imaging flow cytometry with a real-time storage rate beyond 5.9 GB/s. *Cytom. Part A* **105**, 713–721 (2024). 10.1002/cyto.a.24854 38842356

[71] Liu, X. et al. An optimized PDMS microfluidic device for ultra-fast and high-throughput imaging flow cytometry. *Lab a Chip* **23**, 3571–3580 (2023). 10.1039/d3lc00237c 37401791

