# Supplementary material for: Imaging flow cytometry with a real-time throughput beyond 1,000,000 events per second
Source: Light Sci Appl. 2025 Feb 10;14:76. doi: 10.1038/s41377-025-01754-9 (PMC11808109; doi:10.1038/s41377-025-01754-9)
Supplement: Supplementary file 1 — Supplementary Information for Imaging flow cytometry with a real-time throughput beyond 1,000,000 events per second [file 41377_2025_1754_MOESM1_ESM.docx]

**Supplementary Information for**

Imaging flow cytometry with a real-time throughput beyond 1,000,000 events per second

Jiehua Zhou,^1,^^†^ Liye Mei,^1,2, †^ Mingjie Yu,^1^ Xiao Ma,^1^ Dan Hou,^1^ Zhuo Yin,^1^ Xun Liu,^1,3^ Yan Ding,^1^ Kaining Yang,^1^ Ruidong Xiao,^1^ Xiandan Yuan,^1,4^ Yueyun Weng,^1^ Mengping Long,^1,5^ Taobo Hu,^1,6^ Jinxuan Hou,^7^ Yu Xu,^8^ Liang Tao,^9^ Sisi Mei,^9^ Hui Shen,^10^ Yaxiaer Yalikun,^3^ Fuling Zhou,^10^ Liang Wang,^11,*^ Du Wang^1,*^ Sheng Liu^1^ and Cheng Lei^1,12,13,*^

^1^ The Institute of Technological Sciences, Wuhan University, Wuhan, 430072, China

^2^ School of Computer Science, Hubei University of Technology, Wuhan, 430068, China

^3^ Division of Materials Science, Nara Institute of Science and Technology, Takayama-cho,8916-5, Japan

^4^ School of Science, Hubei University of Technology, Wuhan, 430068, China

^5^ Department of Pathology, Peking University Cancer Hospital, Beijing, 100142, China

^6^ Department of Breast Surgery, Peking University People's Hospital, Beijing, 100044, China

^7^ Department of Thyroid and Breast Surgery, Zhongnan Hospital, Wuhan University, Wuhan, 430071, China

^8^ Department of Radiation and Medical Oncology, Zhongnan Hospital, Wuhan University, Wuhan, 430071, China

^9^ People' Hospital of Anshun City Guizhou Province, Anshun, 561000, China

^10^ Department of Hematology, Zhongnan Hospital, Wuhan University, Wuhan, 430071, China

^11^ National Engineering Laboratory for Next Generation Internet Access System, School of Optics and Electronic Information, Huazhong University of Science and Technology, Wuhan 430074, China

^12^ Suzhou Institute of Wuhan University, Suzhou, 215000, China

^13^ Shenzhen Institute of Wuhan University, Shenzhen, 518057, China

*^†^These authors contributed equally to this work.*

^*^ Corresponding authors: [hustwl@hust.edu.cn](mailto:hustwl@hust.edu.cn) (L.W.), [wangdu@whu.edu.cn](mailto:wangdu@whu.edu.cn) (D.W.), [leicheng@whu.edu.cn](mailto:leicheng@whu.edu.cn) (C.L.)

***S.1*** ***Synchronization between OTS imaging and real-time processing***

As shown in Fig. S1, we input the ~80 MHz pulse synchronization signal output by the Vitara Modelocked Ti:S laser into the Valon Technology 5009a frequency synthesizer (A low-pass filter is integrated), which can generate a 2.56 GHz clock signal after selecting the frequency multiplying factor of 32. An oscilloscope was used to simultaneously observe the image signal and the 2.56 GHz clock signal, with either signal selected as the trigger source. Once the waveforms of the two signals were synchronized and remained stable relative to each other, the clock signal was connected from either Source 1 or Source 2 output of the Valon 5009 frequency synthesizer to the CLK input of the ADQ7, while the image signal was connected to the X input of the ADQ7. All signals were transmitted through SMA interface connections.


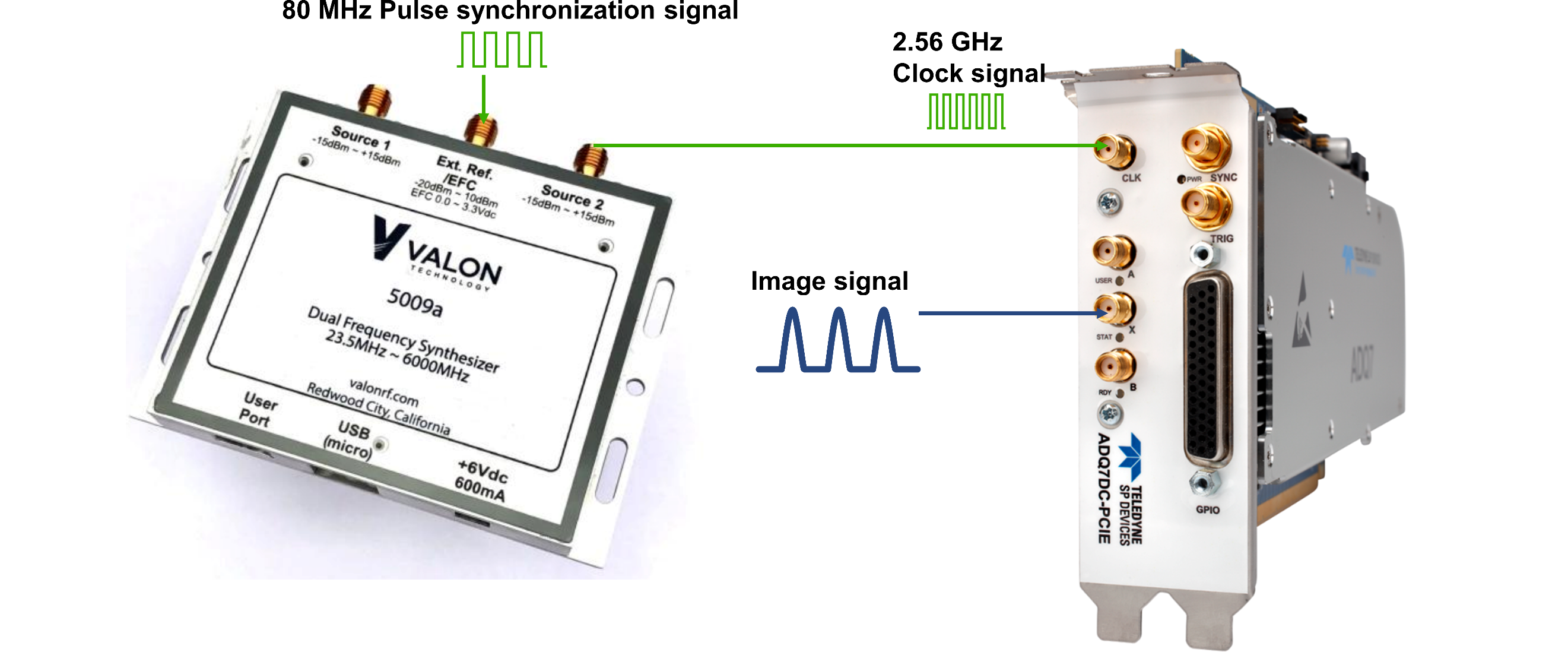


**Fig. S1.** The generation of synchronous clock signals and the input connections to the ADQ7.

***S.2 T******he theoretical analysis of redundancy data removal in OTS-IFC***

As shown in Fig. S2, optofluidic time-stretch imaging generates significant redundant data. We categorize this redundant data into four parts and provide a theoretical analysis of the data reduction proportion (DRP) achieved by applying different approaches to remove redundancy in each part. Correspondingly, we respectively illustrate DRP by taking our system as an example. First of all, the noise signal between adjacent pulses does not carry any image information, which is redundant data that can be discarded. The DRP is determined by pulse duty ratio and can be [approximatively](javascript:;) evaluated as:

|  | $P_{DRP1}=1-t/T$ | (1) |
| --- | --- | --- |

where $T$ is the pulse repetition period of 12.5 ns, $t$ is the pulse width of 6.4 ns in our experimental system; we need to consume two clock periods (6.4 ns) to cover a pulse width, thus achieving the DRP of 1/2 in our synchronous system.

Secondly, the data between cell and cell carry useless information, i.e., the pulse betwixt frames is superfluous data that can be discarded. In other words, we are only concerned about the region of interest (ROI) that includes cells. The DRP is estimated as follows:

|  | $P_{DRP4}=1-\emptyset_{acc}/\emptyset$ | (2) |
| --- | --- | --- |

where $\emptyset_{acc}$ is the actual event rate of cells, and $\emptyset$ is the theoretical maximum event rate. By defining the flow direction cell field of view (FOV) as 100 µm per frame, the maximum event rate $\emptyset$ can be calculated as:

|  | $\emptyset=v/(100um)$ | (3) |
| --- | --- | --- |

where $v$ is the practical linear velocity of cell flow in the microfluidic channel. For example, if $v$ is 5 m s^-1^, the maximum cell event rate $\emptyset$ derived from Eq. (3) is 50,000 events per second (eps), and when the actual cell event rate is 10,000 eps, the DRP can be calculated as 4/5.

Thirdly, 16-bit quantization of the image signal is unnecessary, and 8-bit quantization is sufficient to show the image's detail after removing the background on FPGA. According to the dynamic range of the image signal, any consecutive 8 bits of the 16-bit data can be selected as the output. The DRP is straightforwardly given as:

|  | $P_{DRP3}=1/2$ | (4) |
| --- | --- | --- |

Finally, when cells do not reach the upper limit of linear velocity in the microfluidic channel (which depends on the pulse repetition rate and fabrication of the microfluidic channel), two or even dozens of adjoining pulses may convey identical image 1D (line) information, which is tautological data that can be discarded. The corresponding DRP is described as follows:

|  | $P_{DRP2}=1-v/v_{max}$ | (5) |
| --- | --- | --- |

where $v$ is the practical linear velocity of cell flow in the microfluidic channel, $v_{max}$ is the theoretical maximum linear velocity of a cell that maintains adequate pixel to ensure image quality, without accounting for the tolerance of cells and the microfluidic channel to hydrodynamic pressure. The number of pulses contained in each frame can be calculated as:

|  | $N=1/(T\emptyset)$ | (6) |
| --- | --- | --- |

We use a Ti: Sapphire femtosecond pulse laser as the optical source, and its center wavelength, bandwidth, and pulse repetition rate are 800 nm, 40 nm, and 80 MHz, respectively; the axial resolution of the system can be roughly calculated as 0.8 µm. Therefore, to ensure sufficient sampling, the 100 µm field of view (FOV) must contain at least 125 pulses, with a recommended minimum of 250 pulses to satisfy the Nyquist sampling theorem. Consequently, $N>125$, so $v_{max}$ can be calculated as 64 m s^-1^. When the practical linear velocity $v$ is 8 m s^-1^, the $P_{DRP2}$ is calculated as 7/8 using Eq. (5).

The DRP for the four approaches illustrated above is summarized in Table S1. Depending on the system configuration and the experimental situation, a total DRP greater than 18/25 must be applied for the continuous data transfer, resulting in a data rate (DR) of less than 5.6 GB s^-1^.

| **Table S1. Summary of Four Approach** | | | | |
| --- | --- | --- | --- | --- |
| Approach | Noise between pulses | Useless data between cells | 16-bit to 8-bit | Oversampling pulses  in cell |
| DRP | $1-t/T$ | $1-$ $\emptyset_{acc}$/ $\emptyset$ | 1/2 | $1- v/v_{max}$ |


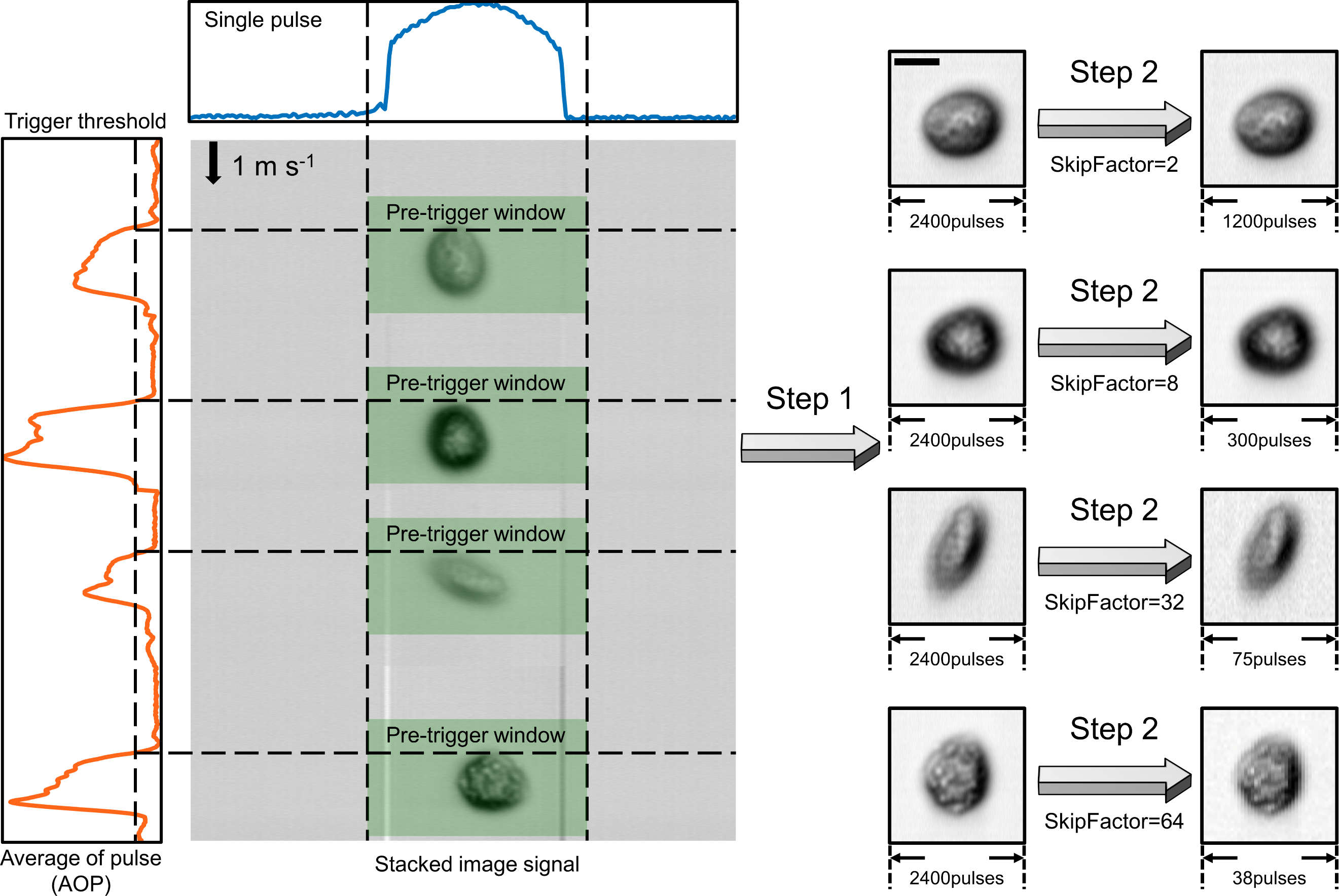


**Fig. S2.** The redundancy in time-stretch imaging and the principle of cell trigger and analysis. Cell image crop and reduce the number of pulses in each cell image to discard the redundancy. Calculate the average of each pulse to engender the AOP signal for cell trigger and image analysis. Scale bars: 10 µm.

***S.3 Comparison with the CMOS-based imaging flow cytometry***

To demonstrate the superiority of our proposed OTS-IFC system, we compared the performance of OTS-IFC and a homemade CMOS-based IFC by acquiring Hela cell images at different flow rates. The CMOS-based IFC setup included a white LED light source, a 20× objective lens (NA: 0.60), and a CMOS camera (Cyclone-2-2000), operating at a frame rate of 8,000 FPS with an exposure time of 2 µs. As illustrated in Fig. S3, OTS-IFC consistently captured sharp and detailed images even as the flow rate increased from 5 m s^-1^ to 15 m s^-1^, with no noticeable artifacts at higher speeds. The cell outlines and internal structures remained well-defined. In contrast, the CMOS-based IFC produced clear images only at lower flow rates. At 0.1 m s^-1^, the cell boundaries and internal features were distinctly visible. However, as the flow rate increased to 2 m s^-1^, the cell outlines became blurry, and fine details were lost. At flow rate of 5 m s^-1^, the images were heavily distorted with motion blur, rendering cell details indistinguishable. These results highlight the robustness of OTS-IFC in maintaining imaging quality at high flow rates compared to conventional CMOS-based systems.


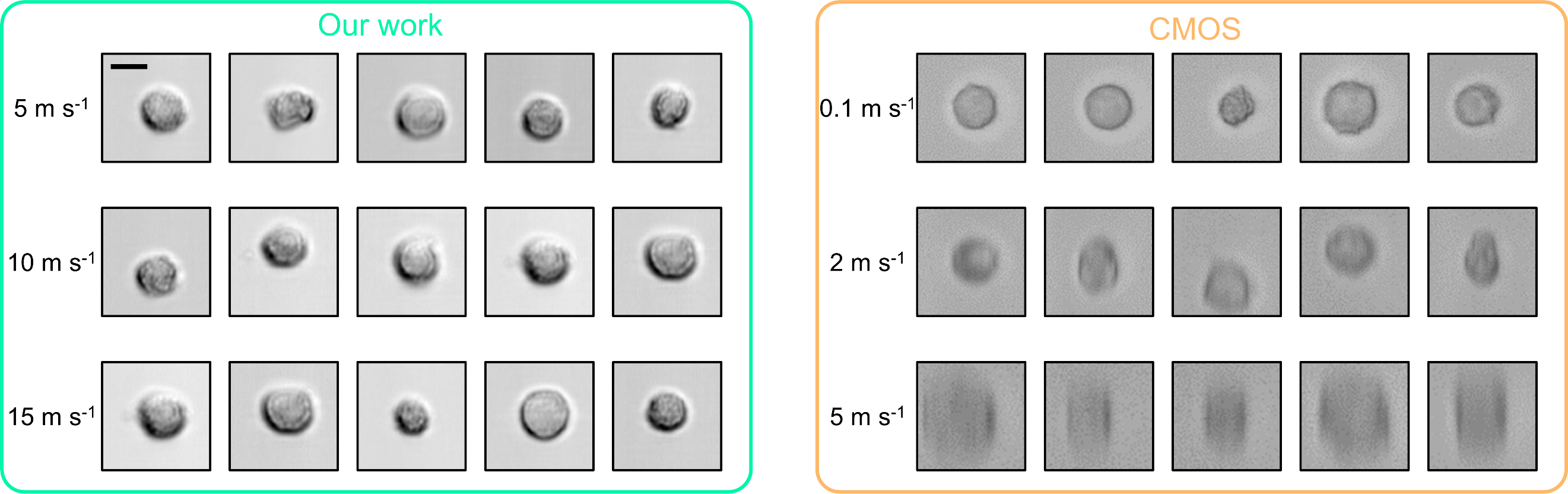


**Fig. S3.** Hela cell images acquired by OTS-IFC and CMOS-based IFC at different flow rates. Scale bars: 10 µm.

***S.4*** ***The influence of the focusing effect on imaging quality in our OTS-IFC***

As shown in Fig. S4, we systematically adjusted the position of the microfluidic chip to evaluate the impact of the focusing effect on imaging quality. At the optimal focal plane (z = 0 µm), the event rate was maximized, yielding the clearest and most detailed images of the cells. When the microfluidic chip was raised by 10 µm (z = 10 µm), nearly all captured images were out of focus, resulting in significant loss of detail and introducing calculation errors in cell size calculation. In contrast, when the chip was lowered by 10 µm from the optimal position (z = -10 µm), most cells remained focused at z = 0 µm, resulting in a significantly reduced likelihood of triggering events and a marked decline in the event rate. This observation highlights the critical importance of precise positioning of the microfluidic chip to ensure optimal imaging quality and reliable data acquisition and analysis.


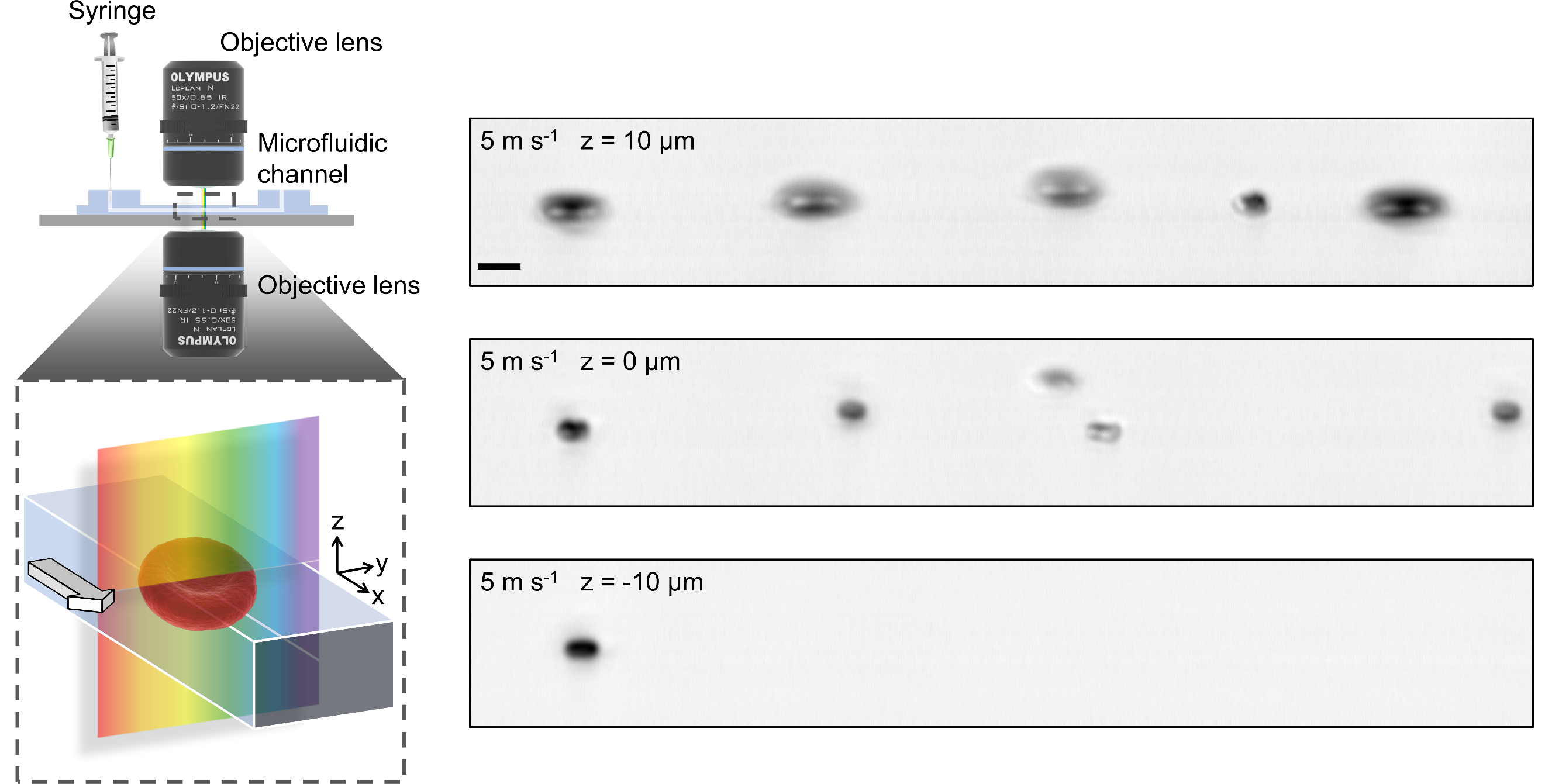


**Fig. S4.** The influence on imaging quality was evaluated by adjusting the position of the microfluidic chip around the optimal imaging focal plane (z = 0 µm). Scale bars: 10 µm.

***S.5 The details of the model and*** ***training process***

The cell size distribution of colorectal tumor and normal tissue samples is illustrated in Fig. S5(a). Following gating with a 10 µm threshold, the filtered image library of these tissue samples is shown in Fig. S5(b). Further details about the CNN architecture employed for classification are provided in Table S2.


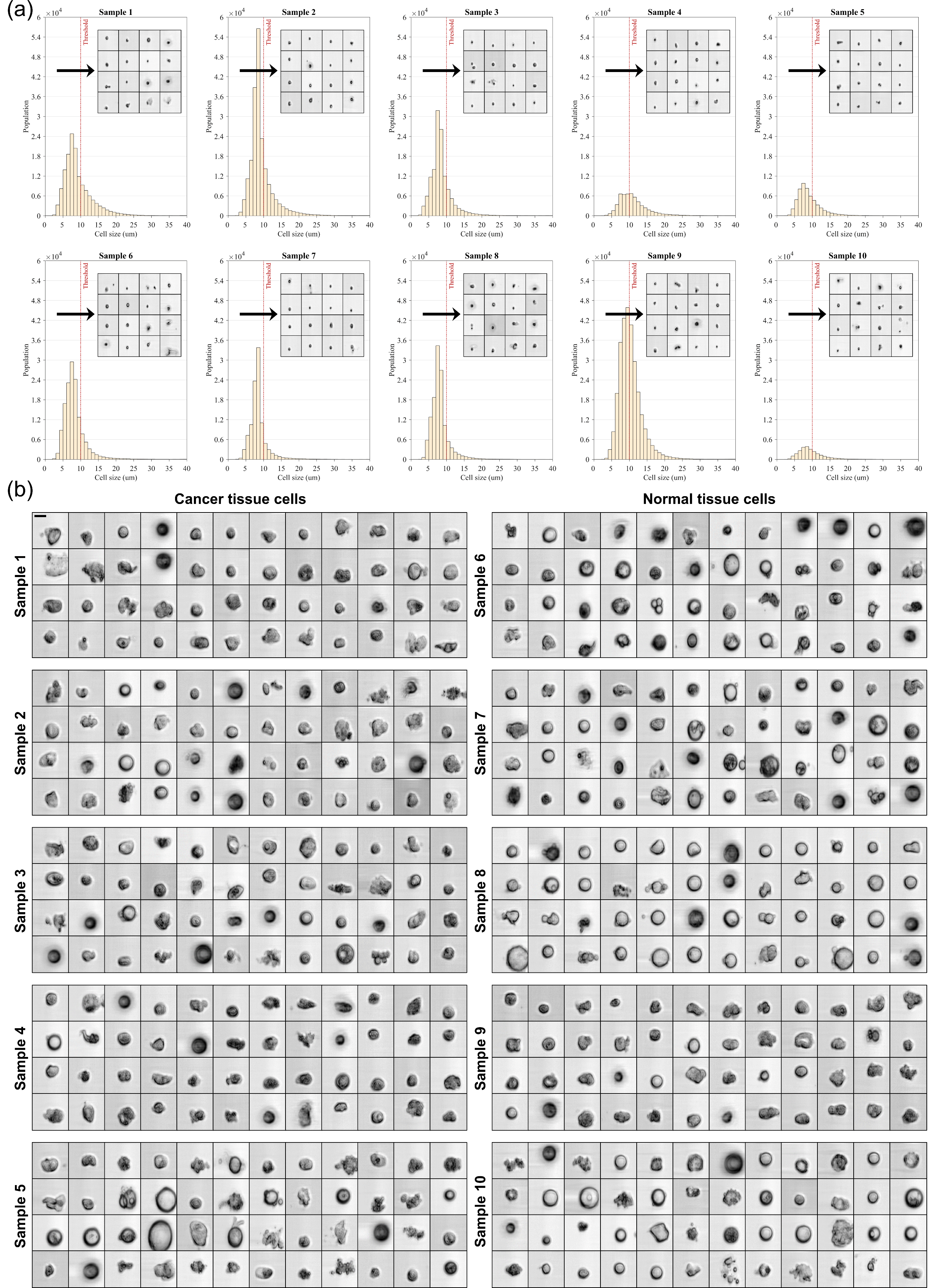


**Fig. S5.** (a) The cell size distribution of different samples of colorectal tumor and normal tissue. (b) Image library of different samples of colorectal tumor and normal tissue after filtering by gating threshold. Scale bars: 10 µm.

**Table S2. The architecture details of the CNN.**

| **Layer** | **Kernel size** | **Stride** | **Padding** | **Input channel** | **Feature map size** | **Activation function** |
| --- | --- | --- | --- | --- | --- | --- |
| Conv1 | 7×7 | 2 | 3 | 128 | 64×184×184 | ReLU |
| CBAM1 | - | - | - | 64 | 64×184×184 | Sigmoid |
| Max pooling | 3×3 | 2 | 1 | 64 | 64×92×92 | - |
| Resnet Block1 | 3×3 | 1, 1  1, 1 | 0 | 64, 64  64, 64 | 64×92×92 | ReLU |
| Resnet Block2 | 3×3 | 2, 1  1, 1 | 0 | 64, 128  128, 128 | 128×46×46 | ReLU |
| Resnet Block3 | 3×3 | 2, 1  1, 1 | 0 | 128, 256  256, 256 | 256×23×23 | ReLU |
| Resnet Block4 | 3×3 | 2, 1  1, 1 | 0 | 256, 512  512, 512 | 512×12×12 | ReLU |
| CBAM2 | - | - | - | 512 | 512×12×12 | Sigmoid |
| Avg pooling | 12×12 | 2 | - | 512 | 512×1×1 | - |
| FC | - | - | - | 512 | 3 | Softmax |

***S.6 The PNRP algorithm of reconstruction of cell images for asynchronous system***

It is worth noting that all discard redundancy methods discussed above are suitable for both synchronous and asynchronous configuration systems. However, the construction of the cell images for asynchronous systems needs to digitally shift and stack the 1D image signals and is tremendously dependent on the original periodicity of the data. To meet the continuous data transfer requirement, we must wipe off redundant data in raw data, such as the noise among adjacent pulses and the adjoining pulses that convey identical information. As shown in Fig. S6, these two processes will corrupt the data's original periodicity without synchronous configuration. For completely reconstructing the processed data into cell images, the traditional approach calculates the correlations between adjacent pulses to stack the 1D image signal. Nevertheless, that paradigm will construct the distorted image while operating the data that discards tautological pulse because of the smaller correlations between adjoining pulses of the processed data. As illustrated in Fig. S6, for correctly constructing the cell images, we propose the pseudo-noise reconstruction period (PNRP) algorithm to retrieve the original periodicity of data unerringly via the feature of FPGA processing data. On account of the characteristic of FPGA 32 parallel processing, FPGA will manipulate a data block containing 32 samples at each clock. That is to say, the data discarded between adjacent pulses in the processed data must be integer multiples of 32. According to adding 32×k pseudo-noise points between adjacent pulses in the processed data, the recovery data will have the consistent periodicity as the original data, where k meets the condition that the noise points after adding pseudo-noise points are closest to the noise points in the original data. It is worth noting that the PNRP algorithm is crucial for continuous data transfer for the asynchronous system because it ensures that all frame data can be completely reconstructed into a cell image at that configuration.


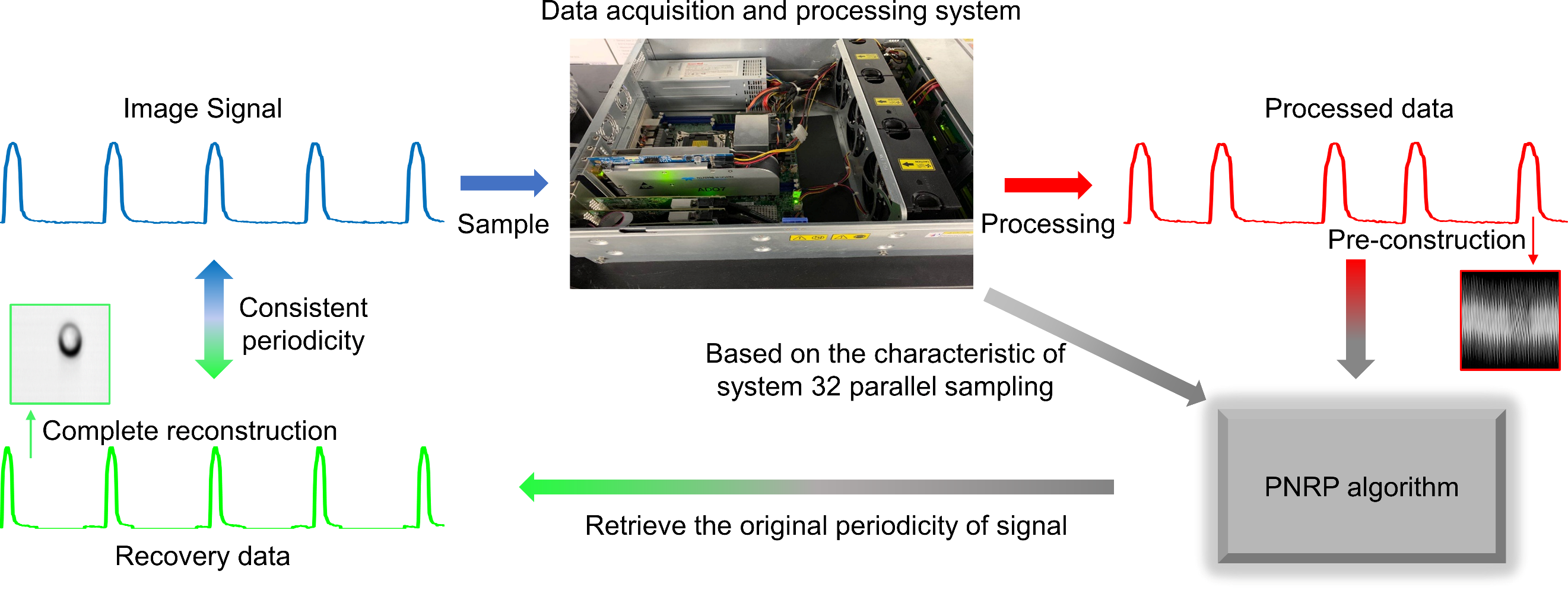


**Fig. S6.** The PNRP algorithm of reconstruction of cell images for the asynchronous system. The original periodicity of the data will be corrupted after processing by the asynchronous system, and the recovery data will have the consistent periodicity as the original data by adding pseudo-noise points between adjacent pulses in the processed data.

***S.7 The data acquisition and processing performance for synchronous and asynchronous configurations***

We compare the data acquisition and processing performance for synchronous and asynchronous configurations. Firstly, we acquire the data generated in case cervical cells flow in the microfluidic channel at a flow speed of 5 m s^-1^ by the system without synchronous configuration. Using the algorithm that we built in the FPGA to trigger each frame of cells and discard redundancy for each frame of cells, we achieved a cell detection throughput of ~10,000 cells s^-1^ at an average DR of only 226.6 MB s^-1^ (acting a skip factor of 4). The resultant practical throughput of cells is calculated as 10,315 fps × 120 pulses × 96 pixels (16-bit) = 226.6 MB s^-1^. Fig. S7(a) shows the comparison of our cytometry data acquisition and processing performance before and after deploying the algorithm in the above experiment condition. The figure shows that each image contains at least one complete cell, and DR reduces from 1188.6 MB s^-1^ to 226.6 MB s^-1^ at the FPS of 10,315 after development. Since the data size of each image (DSI) reduces from 118 KB to 22.5 KB, the time required for image reconstruction (TRIR) in the back end reduces from 554 ms to 298 ms even if the image construction process that digitally shifts and stacks the pulse adds the PNRP algorithm. This indicates that the time required to perform subsequent large-scale single-cell analysis will be reduced accordingly. Secondly, we also acquire the data generated in case whole-blood cells flow in the microfluidic channel at a flow speed of 10 m s^-1^ by the system with synchronous configuration, which achieves a cell detection throughput of ~100,000 cells s^-1^ at a DR of only 803 MB s^-1^ (acting a skip factor of 2). The resultant practical throughput of cells is calculated as 109,670 fps × 120 pulses × 64 pixels (8-bit) = 803.2 MB s^-1^. The comparison of data processing and acquisition performance before and after deploying the algorithm on FPGA in the above experiment condition is shown in Fig. S7(b). Similarly, the figure shows that each image contains at least one complete cell, and DR reduces from 6425.9 MB s^-1^ to 803.2 MB s^-1^ at FPS of 109,670 after development, below the bottleneck limit of the PCIe x8 Gen3 interface DR of 5.6 GB s^-1^, thus realize real-time data transfer and continuous high throughput cell detection. The DSI reduces from 60 KB to 7.5 KB, so the TRIR of directly stacks pulse in the back end reduces from 42 ms to 31 ms.


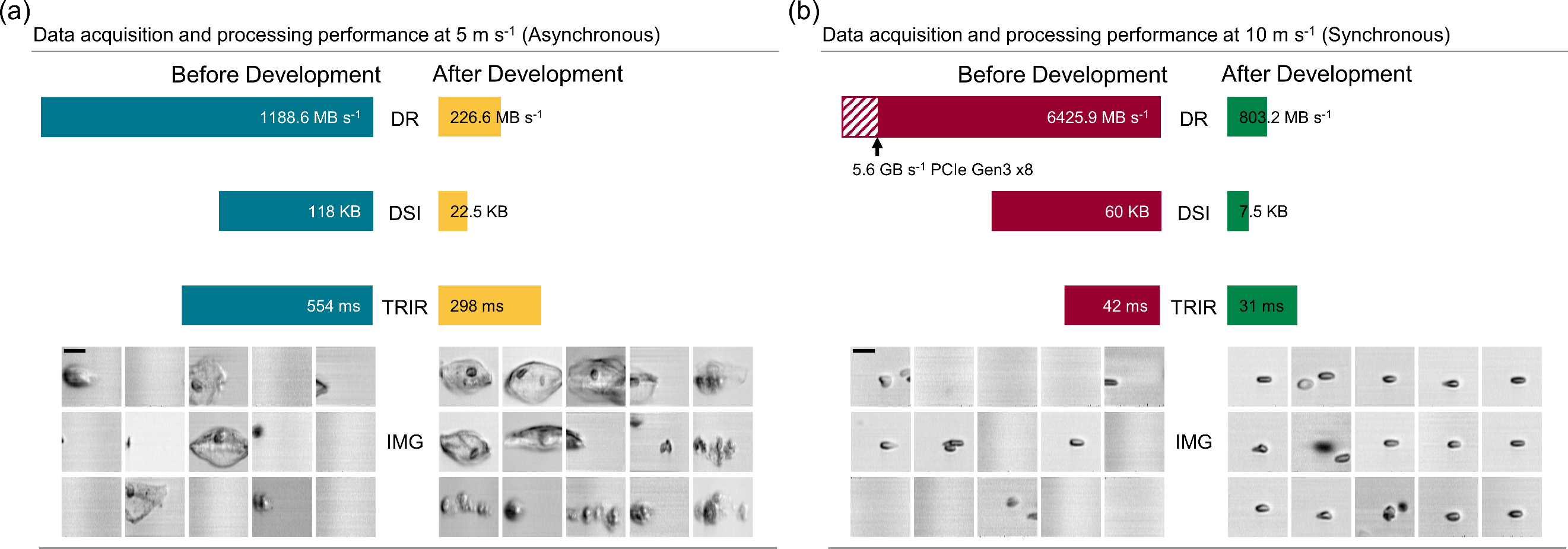


**Fig. S7.** Compare the data acquisition and processing performance for synchronous and asynchronous configurations. (a) Data acquisition and processing performance of cervical cells that flow at 5m s^-1^ (asynchronous configuration). (b) Data acquisition and processing performance of whole-blood cells that flow at 10m s^-1^ (synchronous configuration). DR: data rate. DSI: data size of each image. TRIR: the time required for image reconstruction (Image visualization). IMG: cell image. Scale bars: 10 µm.
